# A Highly Electrostrictive Salt Cocrystal and the Piezoelectric Nanogenerator Application of Its 3D-Printed Polymer Composite

**DOI:** 10.1021/acsami.4c03349

**Published:** 2024-05-10

**Authors:** Supriya Sahoo, Rishukumar Panday, Premkumar Kothavade, Vijay Bhan Sharma, Anirudh Sowmiyanarayanan, Balu Praveenkumar, Jan K. Zaręba, Dinesh Kabra, Kadhiravan Shanmuganathan, Ramamoorthy Boomishankar

**Affiliations:** † Department of Chemistry, Indian Institute of Science Education and Research Pune, Dr. Homi Bhabha Road, Pune 411008, India; ‡ Centre for Energy Science, Indian Institute of Science Education and Research Pune, Dr. Homi Bhabha Road, Pune411008, India; § Polymer Science and Engineering Division, CSIR-National Chemical Laboratory, Dr. Homi Bhabha Road, Pune 411008, India; ∥ Academy of Scientific and Innovative Research (AcSIR), Ghaziabad 201002, India; ⊥ Department of Physics and Center for Research in Nanotechnology and Sciences, Indian Institute of Technology, Mumbai 400076, India; # PZT Centre, Armament Research and Development Establishment, Dr. Homi Bhabha Road, Pune 411021, India; ∇ Institute of Advanced Materials, Wrocław University of Science and Technology, Wrocław 50-370, Poland

**Keywords:** cocrystals, ferroelectricity, piezoelectricity, 3D printing, energy harvesting

## Abstract

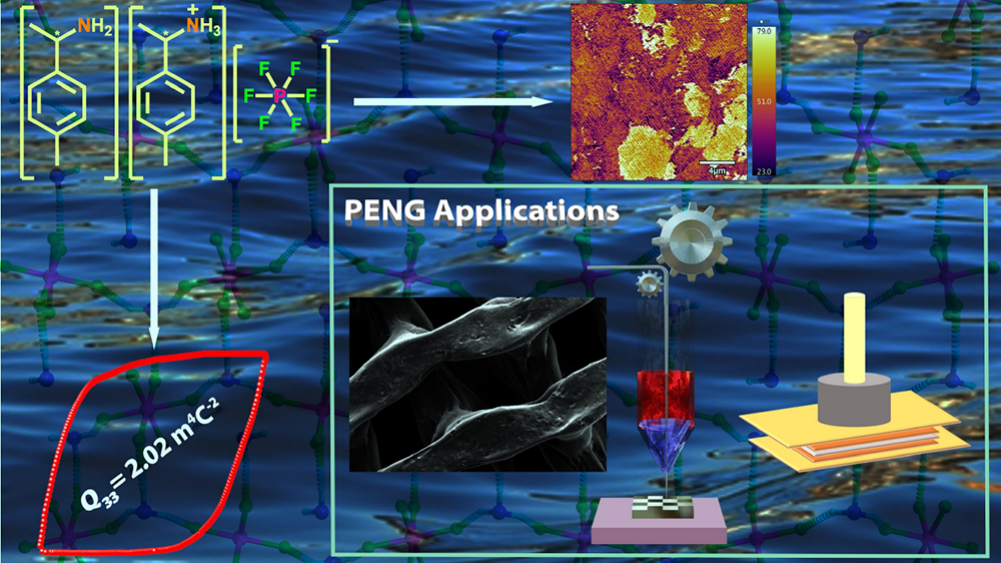

Ionic cocrystals with hydrogen bonding can form exciting materials with enhanced optical and electronic properties. We present a highly moisture-stable ammonium salt cocrystal [CH_3_C_6_H_4_CH(CH_3_)NH_2_][CH_3_C_6_H_4_CH(CH_3_)NH_3_][PF_6_] (**(*****p*****-TEA)(*****p*****-TEAH)·PF**_**6**_) crystallizing in the polar monoclinic *C*2 space group. The asymmetry in **(*****p*****-TEA)(*****p*****-TEAH)·PF**_**6**_ was induced by its chiral substituents, while the polar order and structural stability were achieved by using the octahedral PF_6_^–^ anion and the consequent formation of salt cocrystal. The ferroelectric properties of **(*****p*****-TEA)(*****p*****-TEAH)·PF**_**6**_ were confirmed through *P–E* loop measurements. Piezoresponse force microscopy (PFM) enabled the visualization of its domain structure with characteristic “butterfly” and hysteresis loops associated with ferro- and piezoelectric properties. Notably, **(*****p*****-TEA)(*****p*****-TEAH)·PF**_**6**_ exhibits a large electrostrictive coefficient (*Q*_33_) value of 2.02 m^4^ C^–2^, higher than those found for ceramic-based materials and comparable to that of polyvinylidene difluoride. Furthermore, the composite films of **(*****p*****-TEA)(*****p*****-TEAH)·PF**_**6**_ with polycaprolactone (PCL) polymer and its gyroid-shaped 3D-printed composite scaled-up device, **3DP-*****Gy***, were prepared and evaluated for piezoelectric energy-harvesting functionality. A high output voltage of 22.8 V and a power density of 118.5 μW cm^–3^ have been recorded for the **3DP-*Gy*** device. Remarkably, no loss in voltage outputs was observed for the **(*****p*****-TEA)(*****p*****-TEAH)·PF**_**6**_ devices even after exposure to 99% relative humidity, showcasing their utility under extremely humid conditions.

## Introduction

Cocrystallization of two or more compounds offers a facile route for the generation of distinctive supramolecular assemblies with a range of applications as innovative materials.^1^ Cocrystal engineering is well known for the creation of new organic materials with extraordinary properties that enable comprehensive studies on their structure–property relationships.^1,2^ In this regard, noncovalent interactions such as π···π stacking, hydrogen bonds, halogen bonds, and ion–dipole interactions play a pivotal role in the design of organic cocrystals.^3−5^ These interactions provide significant stability and directional preference, leading to enhanced physiochemical properties desired for functional organic materials.^6^ Materials possessing ferroelectric, dielectric, and piezoelectric properties are extensively explored as ferroelectric random access memory devices, actuators, wearable electronics, electromechanical transducers, medical devices, robotics, and energy storage materials.^7−16^ Among these, ferroelectric materials possessing piezoelectric characteristics are particularly useful for their ability to convert mechanical stimuli into electrical voltage, making them suitable for use in piezoelectric nanogenerators (PENGs).^17−20^ PENGs have become increasingly important in modern high-tech electronics, particularly for energy harvesting, storage, and dissipation.^21−27^ However, the electrostrictive coefficient (*Q*_33_), the measure of PENG output for any piezoelectric material, is low for traditional ceramic materials (0.034–0.096 m^4^C^–2^) in comparison with organic piezoelectrics such as polyvinylidene difluoride (1.3 m^4^ C^–2^).^28,29^ Hence, single- and two-component organic compounds are attractive candidates for obtaining piezoelectrics with high electromechanical properties.^30−32^ Over the years, a variety of organic molecules, including croconic acid,^33^ diisopropylammonium chloride (DIPAC),^34^ diisopropylammonium bromide (DIPAB),^35^ imidazolium perchlorate (Im-ClO_4_),^36^ [Hdabco]ClO_4_ (dabco = 1,4-diazabicyclo[2.2.2]octane),^37^ MDABCO–NH_4_I_3_ (MDABCO = *N*-methyl-*N′*-diazabicyclo[2.2.2]octonium),^38^ trimethylamine borane (TMAB),^31^ and ^R^MBA-BF_3_ (^R^MBA = ^R^C_6_H_5_CH(CH_3_)NH_2_),^39^ were found to exhibit ferroelectric properties. These compounds can be classified as single-component, two-component and zwitterionic forms. Notably, two-component ionic salts have been recognized for their very good ferro- and piezoelectric properties. Despite this, in many instances, two-component ferroelectrics based on conventional ammonium cations, more often than not, encounter challenges such as low *T*_c_ values and compromised moisture stability due to their typically hygroscopic characteristics. To address this challenge, the adoption of the “Quasispherical theory” approach has yielded stable and high *T*_c_ ferroelectrics with bulky cations that exhibit lower symmetry.^30^ Moreover, moving from ammonium to phosphonium cations has also been shown to improve the stability of ferroelectric materials.^40,41^ This study presents a methodology for synthesizing organic ferroelectric and piezoelectric materials with improved stability. This is achieved by fabricating ionic cocrystals that incorporate organic salts and neutral conformers, leveraging a synergistic blend of ionic and noncovalent bonding interactions.

Herein, we report a ferroelectric ionic cocrystal [CH_3_C_6_H_4_CH(CH_3_)NH_2_][CH_3_C_6_H_4_CH(CH_3_)NH_3_][PF_6_] (**(*****p*****-TEA)(*****p*****-TEAH)·PF**_**6**_), obtained from the combination of an ammonium salt and a neutral amine. The structural asymmetry in this cocrystal is imposed by homochiral (*S*)-*p*-tolylethylamino substituents, while the noncoordinating octahedral PF_6_^–^ anion greatly contributes to the electric field-reversible polar property necessary for the ferroelectric phenomenon. Compound **(*****p*****-TEA)(*****p*****-TEAH)·PF**_**6**_ is insoluble in water and exhibits remarkable moisture stability even at 99% relative humidity (RH) conditions. Ferroelectric *P–E* hysteresis loop measurements on **(*****p*****-TEA)(*****p*****-TEAH)·PF**_**6**_ gave a saturation polarization (*P*_s_) of 0.95 μC cm^–2^ at 298 K, while its direct piezoelectric (*d*_33_) coefficient was found to be 4 pC N^–1^. Remarkably, these values yielded a high electrostriction coefficient (*Q*_33_) value of 2.02 m^4^ C^–2^, which is higher than those of the piezoceramics and close to that of polyvinylidene difluoride (PVDF). Subsequently, the PENG applications of **(*****p*****-TEA)(*****p*****-TEAH)·PF**_**6**_ have been established on its various weight percentage (wt %) composites with biodegradable polycaprolactone (PCL) polymer. The optimal 10 wt % **(*****p*****-TEA)(*****p*****-TEAH)·PF**_**6**_-PCL composite device showed a maximum peak-to-peak open-circuit voltage (*V*_OC-PP_) of 13.1 V and power density of 104.2 μW cm^–3^, respectively. Spurred by its high moisture stability and sizable PENG output characteristics, we prepared the scaled-up device for the 10 wt % **(*****p*****-TEA)(*****p*****-TEAH)·PF**_**6**_-PCL composite using the 3D-printing technique. Notably, the performance of the gyroid-shaped 3D-printed PENG device was found to be nearly doubled with a *V*_OC-PP_ of 22.8 V and the power density rising up to 118.5 μW cm^–3^. 3D-printed PENG devices based on organic composite materials are in the early stages of research, and materials selection for such processes requires thorough optimization to establish the synergy between the organic compound and polymer matrix.^42−44^ These results emphasize that the cocrystallization of two-component charge-separated systems with neutral organic compounds is an effective strategy for obtaining highly stable organic ferroelectrics for next-generation energy harvesting devices.

## Results and Discussion

### Synthesis, Structure, SHG, and Thermal Studies

**Figure 1:**
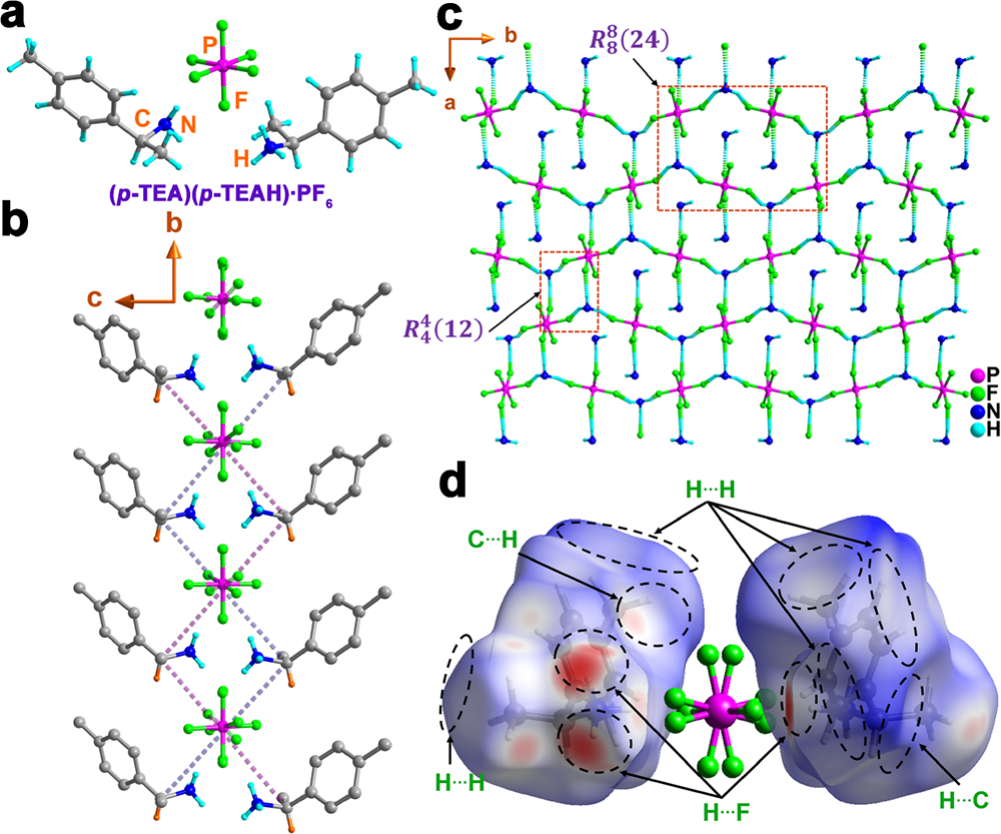
(a) The molecular structure of **(*****p*****-TEA)(*****p*****-TEAH)·PF**_**6**_ at 100 K (the disordered F atoms are omitted for clarity). (b) The view of the zigzag packing of **(*****p*****-TEA)(*****p*****-TEAH)·PF**_**6**_ along the *a*-axis (including the disordered F atoms). (c) The view of two-dimensional hydrogen bonding N–H···F interactions in **(*****p*****-TEA)(*****p*****-TEAH)·PF**_**6**_ along the *ab*-plane. (d) The *d*_norm_ mapped Hirshfeld surface analysis of **(*****p*****-TEA)(*****p*****-TEAH)·PF**_**6**_ (including the disordered F atoms) from its crystal structure at 100 K showing the various interactions present in it.

The cocrystal [CH_3_C_6_H_4_CH(CH_3_)NH_2_][CH_3_C_6_H_4_CH(CH_3_)NH_3_] [PF_6_] (**(*****p*****-TEA)(*****p*****-TEAH)·PF**_**6**_) was synthesized by the treatment of (*S*)-1-(*p*-tolyl)ethylamine (*p*-TEA) with hexafluorophosphoric acid (HPF_6_). The molecular structure of **(*****p*****-TEA)(*****p*****-TEAH)·PF**_**6**_ was solved in the monoclinic *C*2 space group at both 100 and 298 K (Figure 1a,b, Figures S3 and S4, and Table S2). The asymmetric unit of **(*****p*****-TEA)(*****p*****-TEAH)·PF**_**6**_ at both these temperatures was found to contain one *p*-TEA unit and one-half of a disordered PF_6_^–^ anion. The molecular structure contains two *p*-TEA motifs and one PF_6_^–^ anion, in which formally one *p*-TEA is cationic and the other one is neutral. Two fluorine atoms of the PF_6_^–^ ion in the asymmetric unit were positionally disordered over two sites and located in special positions with half-occupancies each. Thus, the charge due to the PF_6_^–^ anion is neutralized by one organic unit (referred to as *p*-TEAH) and the other neutral *p*-TEA motif provides additional H-bonding to the PF_6_^–^ anion. It is to be noted that the *p*-TEAH and *p*-TEA moieties cannot be distinguished crystallographically, and hence, their protons are refined with partial occupancies to provide the accurate charge balance. The packing diagram of **(*****p*****-TEA)(*****p*****-TEAH)·PF**_**6**_ shows the presence of rich H-bonding interactions in which this molecule participates. A close view of the structure reveals that each PF_6_^®^ ion is surrounded by four *p*-TEA/ *p*-TEAH groups while each *p*-TEAH cation is connected with three PF_6_^–^ ions. The cumulative effect of these interactions leads to the formation of a 2D-sheet-like network along the ab-plane. It consists of a series of alternate macrocycles represented by graph set *R*_8_^8^(24) and *R*_4_^4^(12) rings (Figure 1c, Figure S5, and Table S3).^45^ The view of the extended structure, including the disorders, indicates different types of macrocycles with graph set *R*_4_^4^(12) and *R*_4_^4^(12) configurations (Figure S6). The interactions present in compound **(*****p*****-TEA)(*****p*****-TEAH)·PF**_**6**_ were further quantified using Hirshfeld surface analysis of its data collected at 100 K (Figure 1d, Figures S7–S9, and Tables S4 and S5). The analysis revealed multiple types of intermolecular interactions in which the H···F interactions contribute 24.3% to the total intermolecular interactions (Table S5).

The structural composition of **(*****p*****-TEA)(*****p*****-TEAH)·PF**_**6**_ was further examined by using X-ray photoelectron spectroscopy (XPS). The XPS composition analysis showed the existence of the elements C, N, P, and F. The deconvoluted peaks of C(1s) with binding energies at 284.48 and 285.78 eV correspond to the respective C–C/C–H and C–N bonds (Figure S10a).^46^ The N(1s) spectra consist of two peaks with binding energies of 401.08 and 401.88 eV corresponding to N–H groups in NH_2_ and NH_3_^+^ functionalities, respectively (Figure S10b).^47^ Additionally, other binding energy peaks at 685.38 and 133.88 eV were observed in the respective F(1s) and P(2p) spectra (Figure S10c,d).^48^

**Figure 2:**
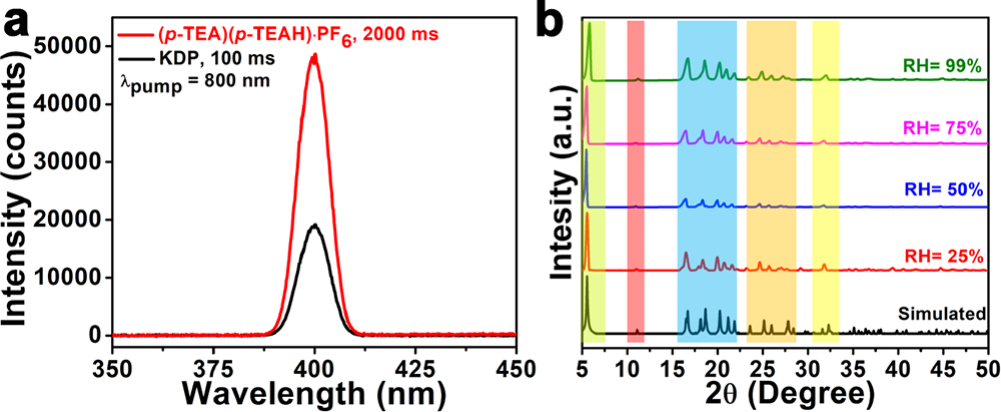
(a) The SHG profile of **(*****p*****-TEA)(*****p*****-TEAH)·PF**_**6**_ and its comparison with standard KDP, obtained upon irradiation with 800 nm femtosecond laser pulses. (b) PXRD profiles of **(*****p*****-TEA)(*****p*****-TEAH)·PF**_**6**_ upon exposure to various humidity conditions showing its high stability.

The acentric structure of **(*****p*****-TEA)(*****p*****-TEAH)·PF**_**6**_ at room temperature was further confirmed by second harmonic generation (SHG) measurement, using a Kurtz–Perry-type setup. The size-graded powder of **(*****p*****-TEA)(*****p*****-TEAH)·PF**_**6**_ was irradiated with an 800 nm, 1 kHz laser with a pulse width of 75 fs. The compound **(*****p*****-TEA)(*****p*****-TEAH)·PF**_**6**_ was observed to emit an SHG with a relative efficiency of 0.1 with respect to the standard potassium dihydrogen phosphate (KDP) sample (Figure 2a).

The phase purity of compound **(*****p*****-TEA)(*****p*****-TEAH)·PF**_**6**_ was confirmed from its powder X-ray diffraction (PXRD) profile. The experimental PXRD peaks matched well with the simulated pattern obtained from the single-crystal diffraction data of **(*****p*****-TEA)(*****p*****-TEAH)·PF**_**6**_ (Figure S11), providing evidence of a phase identity. The instability of ammonium salts in the presence of moisture is a common issue in two-component ferroelectrics.^49^ Hence, to check the moisture stability of **(*****p*****-TEA)(*****p*****-TEAH)·PF**_**6**_, it was exposed to different RH conditions ranging from 25 to 99% and its PXRD profiles were compared with that of the pristine sample (Figure 2b). Also, the Raman spectral analyses were performed for **(*****p*****-TEA)(*****p*****-TEAH)·PF**_**6**_ after exposing it to different conditions of varied humidity and the presence of characteristic Raman active modes associated with C–H (2952, 3024, 3064 cm^–1^), C–C (809 cm^–1^), C–N (1373 cm^–1^), and N–H (2920 cm^–1^) was observed with no significant changes in their peak profiles (Figure S12). Remarkably, compound **(*****p*****-TEA)(*****p*****-TEAH)·PF**_**6**_ is insoluble in water and shows crystalline stability even at 99% RH conditions. This can be attributed to the ionic cocrystalline nature of this material, which involves the hydrophobic PF_6_^–^ unit.

The thermal stability of **(*****p*****-TEA)(*****p*****-TEAH)·PF**_**6**_ was confirmed by thermogravimetric (TGA) analysis, which shows no weight loss up to its decomposition temperature of 550 K (Figure S13). Additionally, differential scanning calorimetry (DSC) measurements showed that the compound does not undergo any phase transition with temperature until its melting point at 490 K. These findings proved that compound **(*****p*****-TEA)(*****p*****-TEAH)·PF**_**6**_ is both thermal and moisture-stable under ambient conditions for an extended period and is capable of retaining its crystallinity even at high RH conditions promising its utility for various applications.

### Ferroelectric, Dielectric, and Piezoelectric Studies

**Figure 3:**
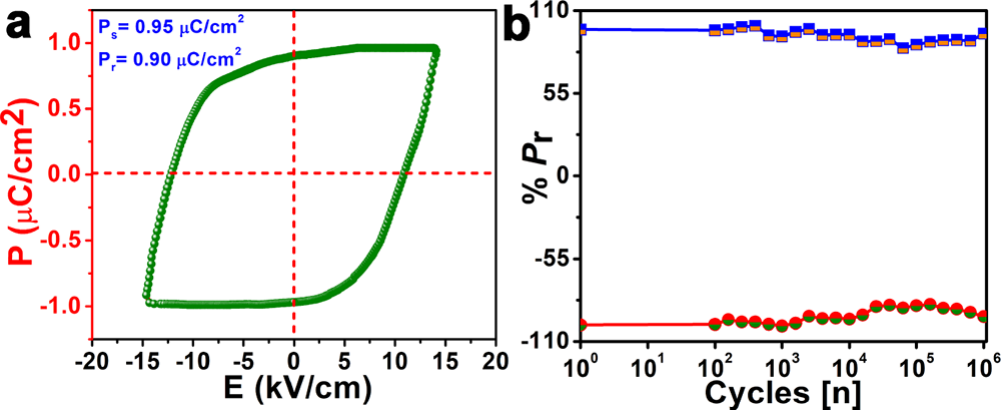
(a) The ferroelectric behavior of **(*****p*****-TEA)(*****p*****-TEAH)·PF**_**6**_ showing the rectangular *P–E* hysteresis loop at 298 K. (b) The fatigue test showing the retention of polarization of **(*****p*****-TEA)(*****p*****-TEAH)·PF**_**6**_ up to 10^6^ cycles at 298 K.

Compound **(*****p*****-TEA)(*****p*****-TEAH)·PF**_**6**_ features the point group symmetry *C*_2_, which belongs to one of the 10 polar point groups compatible with ferroelectric properties. Polarization vs electric field (*P–E*) hysteresis loop measurements were conducted on the powder-pressed pellets of **(*****p*****-TEA)(*****p*****-TEAH)·PF**_**6**_ using a Sawyer–Tower circuit setup at room temperature to investigate its ferroelectric response. A typical rectangular-shaped *P–E* loop was observed for **(*****p*****-TEA)(*****p*****-TEAH)·PF**_**6**_ at 298 K with the saturation polarization (*P*_s_) value of 0.95 μC cm^–2^ comparable to those reported for several ferroelectric materials (Figure 3a and Table S6). The origin of polarization in **(*****p*****-TEA)(*****p*****-TEAH)·PF**_**6**_ can be attributed to the stable charge-separated structure of the compound containing organic ammonium cations and octahedral PF_6_^–^ anions and their involvement in rich nonclassical H···F interactions in the cocrystal. Ferroelectric fatigue measurements on **(*****p*****-TEA)(*****p*****-TEAH)·PF**_**6**_ indicate no notable change in its *P*_s_ values up to 10^6^ cycles, confirming its robust polarization behavior (Figure 3b).

Temperature (*T*)- and frequency (*f*)-dependent permittivity measurements were performed on a compacted pellet of **(*****p*****-TEA)(*****p*****-TEAH)·PF**_**6**_ to obtain further insights into its bulk polarization. These studies reveal the absence of any structural phase transition in **(*****p*****-TEA)(*****p*****-TEAH)·PF**_**6**_, as the real part of the dielectric permittivity (ε*′*) remains constant in the temperature range of 298 to 440 K, close to its melting point. A sizable ε*′* value of 48.0 was noted at 298 K and 1 kHz frequency (Figure S14a). The plot of the dielectric loss factor (tan δ) vs T indicates a low dielectric loss in **(*****p*****-TEA)(*****p*****-TEAH)·PF**_**6**_ supporting its ferroelectric nature (Figure S14b). Similar observations were noted in the ε*′* vs *f* and tan δ vs *f* plots as well (Figures S15).

**Figure 4:**
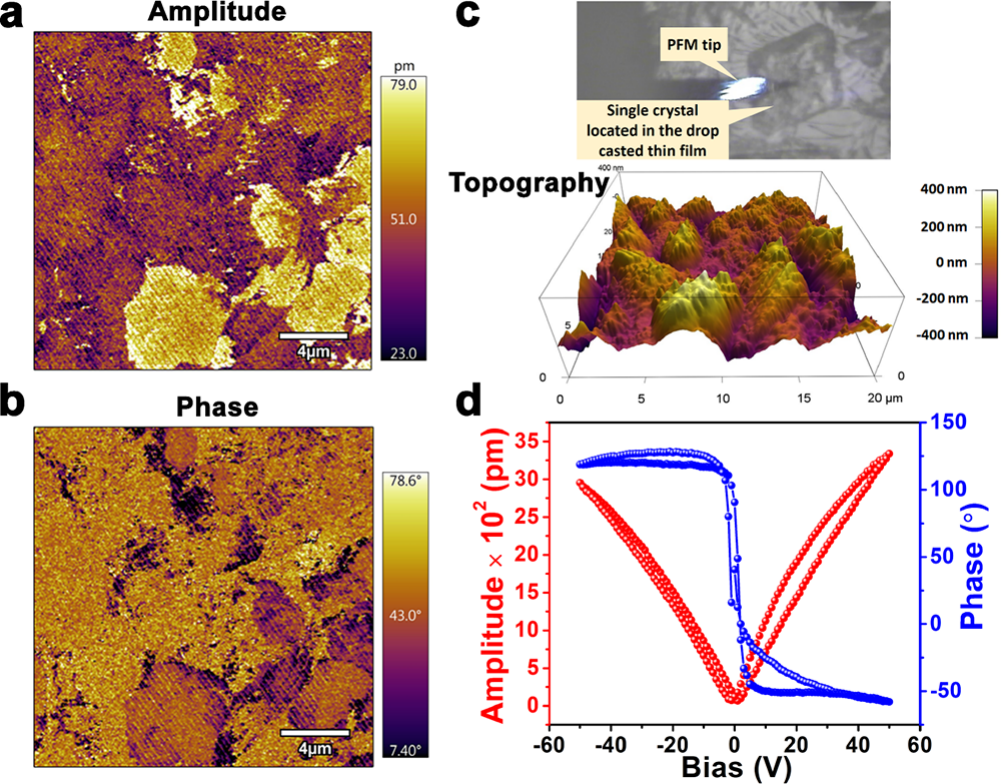
PFM-derived (a) amplitude and (b) phase images of **(*****p*****-TEA)(*****p*****-TEAH)·PF**_**6**_. (c) The visualization of a single crystal of **(*****p*****-TEA)(*****p*****-TEAH)·PF**_**6**_ on the drop-casted thin film along with the PFM tip and its 3D-topography image. (d) The PFM amplitude-bias and phase-bias “butterfly” and “hysteresis” loops of **(*****p*****-TEA)(*****p*****-TEAH)·PF**_**6**_.

The piezoresponse force microscopy (PFM) technique is utilized to investigate the ferro- and piezoelectric properties of **(*****p*****-TEA)(*****p*****-TEAH)·PF**_**6**_ at the nanoscale. Figure 4 shows the PFM studies performed on a single crystal grown on the drop-casted thin film of **(*****p*****-TEA)(*****p*****-TEAH)·PF**_**6**_. Figure 4a is the domain structure showing the amplitude response of **(*****p*****-TEA)(*****p*****-TEAH)·PF**_**6**_ in the vertical-PFM measurements, while Figure 4b is the phase response showing the presence of domains with non-180° orientations. Subsequently, PFM spectroscopic measurements were performed on a single crystal of **(*****p*****-TEA)(*****p*****-TEAH)·PF**_**6**_ located on the surface of the film. These measurements yield the signature butterfly-shaped amplitude bias and the 180° domain switching phase-bias hysteresis loops, which reinforced the piezo- and ferroelectric nature of the material (Figure 4c,d).

Furthermore, piezoelectric measurements performed at an operating frequency of 110 Hz and applied stress of 0.25 N on **(*****p*****-TEA)(*****p*****-TEAH)·PF**_**6**_ using the “Berlincourt” method gave the direct piezoelectric coefficient (*d*_33_) of 4 pC N^–1^. The piezoelectric voltage coefficient (*g*_33_ = *d*_33_/ε_33_, where ε_r_ = ε_33_/ε_0_, ε_0_ = 8.854 × 10^–12^ F m^–1^ and ε_r_ = 11.76 at 1 MHz) was calculated to be of 38.41 × 10^–3^ V m N^–1^. The transduction coefficient (*d*_33_ × *g*_33_), a measure of the power generation ability of any piezoelectric material, was calculated to be 153.64 × 10^–15^ m^3^ J^–1^ for **(*****p*****-TEA)(*****p*****-TEAH)·PF**_**6**_ suggesting its suitability for PENG applications. Moreover, materials with high piezoelectric voltage and electrostrictive coefficients are greatly desired for electromechanical applications via mechanical energy to electrical energy conversion. The electrostrictive coefficient (*Q*_33_ = *g*_33_/2*P*_s_) is calculated to be 2.02 m^4^ C^–2^ for **(*****p*****-TEA)(*****p*****-TEAH)·PF**_**6**_, where *P*_s_ = 0.95 μC cm^–2^. The calculated *Q*_33_ of **(*****p*****-TEA)(*****p*****-TEAH)·PF**_**6**_ is higher than those reported for several ceramic-based piezoelectric materials and is similar to that exhibited by PVDF.^50,51^ Organic polymers with electrostrictive properties, such as polyvinylidene fluoride (PVDF), are recognized for their exceptional electromechanical coupling behavior, making them beneficial for PENG applications. However, it is uncommon to find polymers other than PVDF that demonstrate such high electrostrictive coefficients. Therefore, all-organic materials such as **(*****p*****-TEA)(*****p*****-TEAH)·PF**_**6**_ with a high electrostrictive coefficient (*Q*_33_ = 2.02 m^4^ C^–2^) are advantageous due to their easy synthesis and high moisture stability and are expected to provide significant PENG outputs with optimum mechanical-to-electrical conversion.

### Preparation and Applications of Composite Films

**Figure 5:**
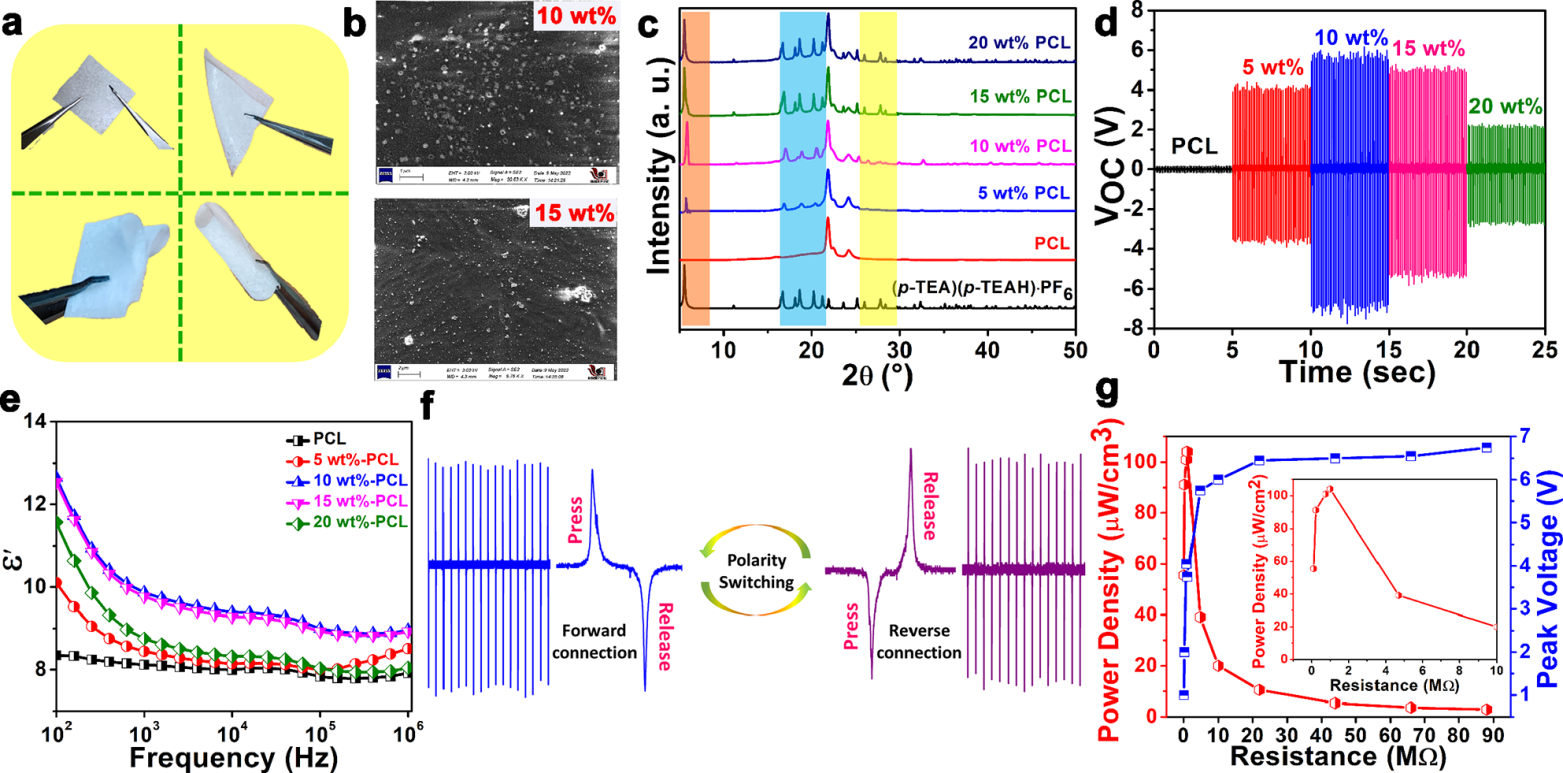
(a) Photographs of a 10 wt % **(*****p*****-TEA)(*****p*****-TEAH)·PF**_**6**_-PCL composite film showing its flexibility, as demonstrated for stretching, bending, twofold bending, and rolling operations. (b) FE-SEM images of the 10 and 15 wt % **(*****p*****-TEA)(*****p*****-TEAH)·PF**_**6**_-PCL composite films. (c) Stacked PXRD profiles of **(*****p*****-TEA)(*****p*****-TEAH)·PF**_**6**_-PCL films and their comparison with the diffraction patterns of as-synthesized **(*****p*****-TEA)(*****p*****-TEAH)·PF**_**6**_. (d) Open-circuit peak-to-peak voltages (*V*_OC-PP_) of **(*****p*****-TEA)(*****p*****-TEAH)·PF**_**6**_-PCL composite devices. The shifted time axis provided here is a guide to the eyes. (e) Frequency-dependent permittivity for all **(*****p*****-TEA)(*****p*****-TEAH)·PF**_**6**_-PCL composite films. (f) The obtained output signals upon forward and reverse connection of the electrode contacts to the oscilloscope. (g) The peak voltage drop and power density (PD) values of the 10 wt % **(*****p*****-TEA)(*****p*****-TEAH)·PF**_**6**_-PCL composite device under different external load resistances. The inset depicts the maximum PD obtained at 1 MΩ resistance.

To further investigate the application of **(*****p*****-TEA)(*****p*****-TEAH)·PF**_**6**_ for piezoelectric energy harvesting applications, its polymer composite films were prepared by incorporating 5, 10, 15, and 20 wt % of **(*****p*****-TEA)(*****p*****-TEAH)·PF**_**6**_ into PCL, a biodegradable polymer (Figure S16). These films were found to exhibit good flexibility for various mechanical motions as shown by their stability for bending and folding operations (Figure 5a). The FE-SEM analyses showed the presence of the piezoelectric crystallites of **(*****p*****-TEA)(*****p*****-TEAH)·PF**_**6**_ within the polymer matrix (Figure 5b and Figures S17 and S18). PXRD analysis confirmed the structural stability and crystallinity of **(*****p*****-TEA)(*****p*****-TEAH)·PF**_**6**_ in the composite films, as evidenced by the presence of characteristic *hkl* peaks of **(*****p*****-TEA)(*****p*****-TEAH)·PF**_**6**_ in all wt % composites (Figure 5c). Also, the Raman spectral analysis confirmed that the structural features of **(*****p*****-TEA)(*****p*****-TEAH)·PF**_**6**_ were preserved in all the **(*****p*****-TEA)(*****p*****-TEAH)·PF**_**6**_-PCL composites (Figure S19). Notably, the Raman active modes associated with neat PCL, C–C (1038 and 1109 cm^–1^), CH_2_ (1418 and 1439 cm^–1^), and C=O (1722 cm^–1^),^52^ as well as that of **(*****p*****-TEA)(*****p*****-TEAH)·PF**_**6**_, C–H (2952, 3024, 3064 cm^–1^), C–C (809 cm^–1^), C–N (1373 cm^–1^), and N–H (2920 cm^–1^), were found to be present in all the composite films.

Furthermore, to validate the mechanical flexibility, stress–strain measurements were performed for neat PCL and those of 5, 10, 15, and 20 wt % of **(*****p*****-TEA)(*****p*****-TEAH)·PF**_**6**_-PCL composites. The yield stress values of 9.8, 8.7, 7.5, and 5.8 MPa were observed for the 5, 10, 15, and 20 wt % composite films, respectively (for neat PCL, it is 12.6 MPa) (Figure S20). The values of Young’s modulus were found to be 158, 144, 101, and 108 MPa for the 5, 10, 15, and 20 wt % composite films, respectively. Upon increasing the content of ferroelectric crystallites in the PCL matrix, a gradual reduction in both the tensile strength and Young’s modulus can be noted, which could be attributed to the crystalline nature of the embedded materials. The observed stress values of all the films at 50% strain (the maximum these devices could experience during the PENG measurements) and the fact that they do not break even after 50% strain indicates the flexible nature of these composite materials. The tensile toughness of the **(*****p*****-TEA)(*****p*****-TEAH)·PF**_**6**_-PCL composite films was determined by calculating the area under the stress–strain curve. The composite films exhibited satisfactory tensile toughness values of 119.1, 90.4, 84.2, and 70.8 MJ m^–3^ for the 5, 10, 15, and 20 wt % **(*****p*****-TEA)(*****p*****-TEAH)·PF**_**6**_ loadings, respectively (Table S7). The neat PCL film resulted in a tensile toughness value of 146.8 MJ m^–3^ agreeing well with the previously reported values (Table S7).^53^

Spurred by the robust characteristics of **(*****p*****-TEA)(*****p*****-TEAH)·PF**_**6**_-PCL films, we set out to test the PENG output performance of their devices. The devices for these studies were obtained by placing adhesive copper tapes as top and bottom electrodes and subsequent encapsulation with Kapton tape for electrical insulation. The peak-to-peak open-circuit voltages (*V*_OC-PP_) of the composite devices with 5, 10, 15, and 20 wt % of **(*****p*****-TEA)(*****p*****-TEAH)·PF**_**6**_-PCL were measured to be 8, 13.1, 10.4, and 5 V, respectively under an external load of 21 N and at an optimized operating frequency of 10 Hz (Figures 5d and Figure S21 and 22). By contrast, for the device made up of neat PCL, a *V*_OC-PP_ of only 0.3 V was measured, indicating that the voltages generated from the composites were due to the piezoelectric nature of **(*****p*****-TEA)(*****p*****-TEAH)·PF**_**6**_.

Evidently, an increase in the *V*_OC-PP_ was observed for the particle loading up to the 10 wt % of **(*****p*****-TEA)(*****p*****-TEAH)·PF**_**6**_ in the composites and shows an abrupt decrease for the 15 and 20 wt % composites. The decrease in voltage outputs for higher concentrations of **(*****p*****-TEA)(*****p*****-TEAH)·PF**_**6**_ in PCL can be attributed to the agglomeration of **(*****p*****-TEA)(*****p*****-TEAH)·PF**_**6**_ crystallites in the polymer matrix, as observed from FE-SEM images of 15 and 20 wt % composites. It is to be noted that the agglomeration of the particles leads to Maxwell–Wagner–Sillars polarization.^54,55^ This phenomenon is clear from the permittivity data measured for these **(*****p*****-TEA)(*****p*****-TEAH)·PF**_**6**_-PCL composites. The frequency dependence of ε*′* values of the composite films initially showed an increasing trend and reached the highest values for the 10 wt % **(*****p*****-TEA)(*****p*****-TEAH)·PF**_**6**_-PCL (ε*′* = 8.9 at 1 MHz) composite (Figure 5e and Figure S23). Afterward, a reduction in ε*′* values for the 15 and 20 wt % composites was observed, indicating a reduction in the effective dipoles in the system.

The piezoelectric nature of the composite device was confirmed by performing a polarity-switching test on the best-performing 10 wt % **(*****p*****-TEA)(*****p*****-TEAH)·PF**_**6**_-PCL device, which showed a maximum *V*_OC-PP_ value of 13 V. Thus, reversing the electrode connections to the oscilloscope during the press and release operations produced signals of equal magnitude and opposite directions, validating the true piezoelectric behavior of the device (Figure 5f and Figure S24). Subsequently, a 4.7 MΩ resistor was employed to measure the voltages across the circuit to determine the calculated output currents. These measurements yielded the peak-to-peak current (*I*_PP_) values of 1.21, 2.34, 1.86, and 1.11 μA for the 5, 10, 15, and 20 wt % **(*****p*****-TEA)(*****p*****-TEAH)·PF**_**6**_-PCL devices, respectively (Figures S25 and S26). Notably, the 10 wt % **(*****p*****-TEA)(*****p*****-TEAH)·PF**_**6**_-PCL composite device yielded the highest *V*_OC-PP_ of 13.1 V and calculated an *I*_PP_ value of 2.34 μA at 4.7 MΩ (Figure S27). In order to assess the effectiveness of composite devices for practical applications, their output peak voltage drops (by default, the voltage generated during the compression cycles) were measured across a range of load resistances, spanning from 100 kΩ to 88 MΩ (Figure S28). The composite device containing 10 wt % **(*****p*****-TEA)(*****p*****-TEAH)·PF**_**6**_-PCL demonstrated the most promising performance, displaying a rise in voltage as the load resistance increased. At 1 MΩ, the peak voltage drop approached a value close to peak open-circuit voltage and ultimately attained saturation at higher resistances (Figure 5g). The PD values of all the **(*****p*****-TEA)(*****p*****-TEAH)·PF**_**6**_-PCL devices at each of the load-resistance-dependent measurements were calculated by the formula *VI*/*Vol.*, where *V* is the peak voltage drop, *I* is the peak current, and *Vol.* represents the volume of the device in cm^3^. The 10 wt % **(*****p*****-TEA)(*****p*****-TEAH)·PF**_**6**_-PCL composite device displayed the highest volume PD value of 104.2 μW cm^–3^ (area PD = 5.2 μW cm^–2^) at 1 MΩ (Figure 5g). Also, the direct piezoelectric coefficient (*d*_33_) value for the highest-performing 10 wt % **(*****p*****-TEA)(*****p*****-TEAH)·PF**_**6**_-PCL composite was measured to be 2 pC N^–1^, validating the piezoelectric nature of the embedded crystallites of **(***p***-TEA)(***p***-TEAH)·PF**_**6**_ inside the polymer matrix.

### 3D-Printed **(*p*-TEA)(*p*-TEAH)·PF_6_**-PCL (**3DP-*Gy***) Device and Its PENG Functionality

**Figure 6:**
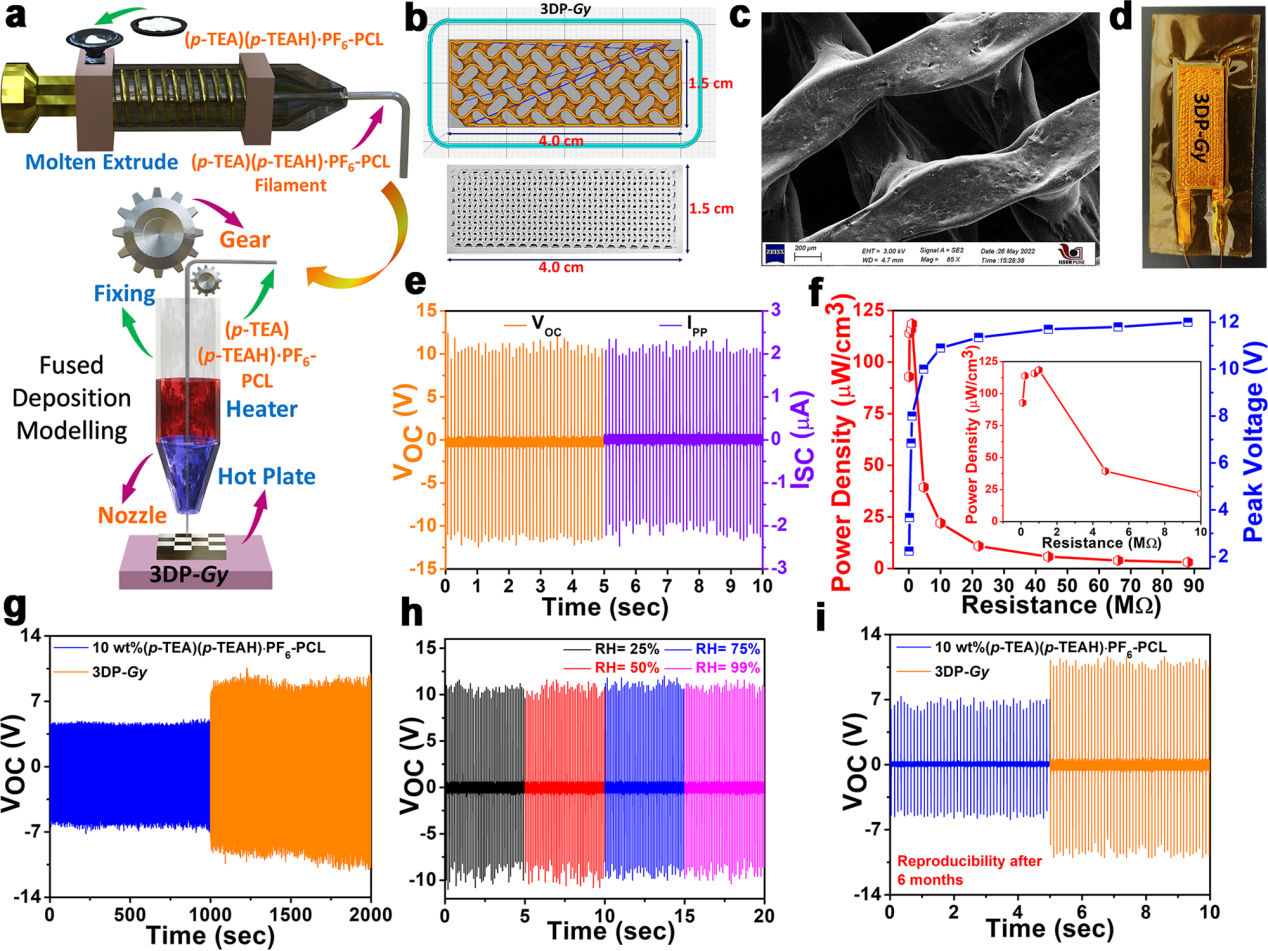
(a) Schematic showing the filament preparation and FDM printing of a **(*****p*****-TEA)(*****p*****-TEAH)·PF**_**6**_-PCL composite slab. (b) Pictures of the computerized and as-made **3DP-*****Gy*** slabs with dimensions. (c) FE-SEM image of the **3DP-*****Gy*** composite slab. (d) Picture of the as-made **3DP-*****Gy*** device. (e) Measured *V*_OC-PP_ and calculated *I*_PP_ for the **3DP-*****Gy*** device (the shifted time axis provided here is a guide to the eye). (f) Calculated power density and voltage drop profile of **3DP-*****Gy*** with a range of load resistances. The inset depicts the maximum PD obtained at 1 MΩ resistance. (g) The cyclic stability tests of 10 wt % **(*****p*****-TEA)(*****p*****-TEAH)·PF**_**6**_-PCL and **3DP-*****Gy*** devices showing the retention of *V*_OC-PP_ up to 10000 cycles. (h) The RH-dependent *V*_OC-PP_ of the **3DP-*****Gy*** device. (i) The reproducibility of *V*_OC-PP_ of 10 wt % **(*****p*****-TEA)(*****p*****-TEAH)·PF**_**6**_-PCL and **3DP-*****Gy*** devices after 6 months.

Encouraged by our recent demonstration of fabricating a gyroid-shaped 3D-printed PCL composite based on a two-component ammonium salt {[(Me)_3_CCH(Me)NH_3_][BF_4_]},^42^ we set out to investigate the scale-up capability of the champion 10 wt % **(*****p*****-TEA)(*****p*****-TEAH)·PF**_**6**_-PCL device via 3D-printing technique. The fused deposition modeling (FDM) method was employed to shape the 10 wt % **(*****p*****-TEA)(*****p*****-TEAH)·PF**_**6**_-PCL composite into a 3D-printed device with gyroidal pores (**3DP-*****Gy***). Using the melt extruder equipment, composite filament wires of 10 wt % **(*****p*****-TEA)(*****p*****-TEAH)·PF**_**6**_**–PCL** for 3D printing were first prepared from the corresponding as-made films (Figure 6a top and Figure S29). The mechanical strengths of the filaments of neat PCL and 10 wt % **(*****p*****-TEA)(*****p*****-TEAH)·PF**_**6**_-PCL were checked to investigate the effect of **(*****p*****-TEA)(*****p*****-TEAH)·PF**_**6**_ particles on the composite filament. Similar observations as that of the thin film-based composites were noted for both the filaments of neat PCL and **(*****p*****-TEA)(*****p*****-TEAH)·PF**_**6**_-PCL with the stress values of 8.31 and 7.45 MPa, respectively, at 50% strain (Figure S30). The values of Young’s modulus were found to be 111 and 125 MPa for neat PCL and **(*****p*****-TEA)(*****p*****-TEAH)·PF**_**6**_-PCL filaments, respectively. The filament was subsequently injected into the FDM 3D printer to produce gyroid-shaped composite slabs (Figure 6a bottom and Figure 6b). The microstructure analysis of the resulting **3DP-*****Gy*** composite revealed a distinct Gy pore and an even dispersion of the crystallites of **(*****p*****-TEA)(*****p*****-TEAH)·PF**_**6**_ throughout the polymer matrix, as observed from its FE-SEM image (Figure 6c and Figure S31). Furthermore, the direct piezoelectric coefficient (*d*_33_) value for the **3DP-*****Gy*** slab was measured to be 2.87 pC N^–1^, validating the intact piezoelectric nature of **(*****p*****-TEA)(*****p*****-TEAH)·PF**_**6**_ inside the Gy slab (Figure S32).

The PENG device based on the **3DP-*****Gy*** composite was accomplished by sticking adhesive copper electrodes as top and bottom contacts and copper wires and Kapton tapes to enclose the device (Figure 6d). The **3DP-*****Gy*** device was used for piezoelectric energy harvesting by applying an external load of approximately 21 N and operating at a frequency of 10 Hz. The *V*_OC-PP_ value of the device was measured to be 22.8 V, which indicates an increase in the output voltage by a value of 9.7 V in comparison with the 10 wt % **(*****p*****-TEA)(*****p*****-TEAH)·PF**_**6**_-PCL composite (Figure 6e left and Figures S33a and S34). The enhancement in the performance of the **3DP-*****Gy*** device could be attributed to the increased device area (from 3.6 to 6 cm^2^), improved dipole alignments (achieved by the 3D printing process), and reduced clamping effect in the 3D-printed composite.

Likewise, the **3DP-*****Gy*** device demonstrated a higher *I*_PP_ of 4.2 μA compared to the corresponding thin film-based device (2.34 μA) (Figure 6e right and Figure S33b). Also, the voltage drop across the **3DP-*****Gy*** device for the various external load resistances showed a trend similar to that observed for the thin-film-based device (Figure S35). The **3DP-*****Gy*** device exhibited a maximum volume PD of 118.5 μW cm^–3^ (area PD = 10.6 μW cm^–2^) at 1 MΩ, which is comparable with many of the reported nanogenerator devices containing all-organic components (Figure 6f). The PENG cycling measurements showed that both the 10 wt % **(*****p*****-TEA)(*****p*****-TEAH)·PF**_**6**_-PCL and **3DP-*****Gy*** devices maintained consistent voltage outputs beyond 10,000 cycles, emphasizing their excellent durability with no signs of device degradation (Figure 6g).

As the compound **(*****p*****-TEA)(*****p*****-TEAH)·PF**_**6**_ is stable in highly humid environments, we tested the PENG output performance of both the 10 wt % **(*****p*****-TEA)(*****p*****-TEAH)·PF**_**6**_-PCL and **3DP-*****Gy*** devices after subjecting them to various RH conditions. Remarkably, the *V*_OC-PP_ value obtained from these devices remained uniform at all RH conditions, ranging from 25 to 99% (Figure 6h and Figure S36). The output voltages for both these devices were tested after 6 months of storage under open air (Figure 6i). Remarkably, no significant drop in *V*_OC-PP_ was observed, even after this extended period. This highlights the exceptional long-time stability of the prepared 10 wt % **(*****p*****-TEA)(*****p*****-TEAH)·PF**_**6**_-PCL and **3DP-*****Gy*** devices. These observations indicate that **(*****p*****-TEA)(*****p*****-TEAH)·PF**_**6**_-PCL devices are compatible with scaling-up processes and are highly suitable for PENG applications under diverse environmental conditions. The sizable PENG outputs derived from both 10 wt % **(*****p*****-TEA)(*****p*****-TEAH)·PF**_**6**_-PCL and **3DP-*****Gy*** devices can be attributed to the inherent piezoelectric nature of the ferroelectric **(*****p*****-TEA)(*****p*****-TEAH)·PF**_**6**_ crystallites with polarizable dipoles embedded in the PCL matrix. Furthermore, the impedance properties of both the top-performing 10 wt % **(*****p*****-TEA)(*****p*****-TEAH)·PF**_**6**_-PCL and **3DP-*****Gy*** composites were analyzed. Both samples exhibited similar frequency-dependent behavior, with the impedance increasing as the frequency decreased (Figure S37 and Table S8). The increase in impedance at lower frequencies is a typical characteristic of dielectric materials.

**Figure 7:**
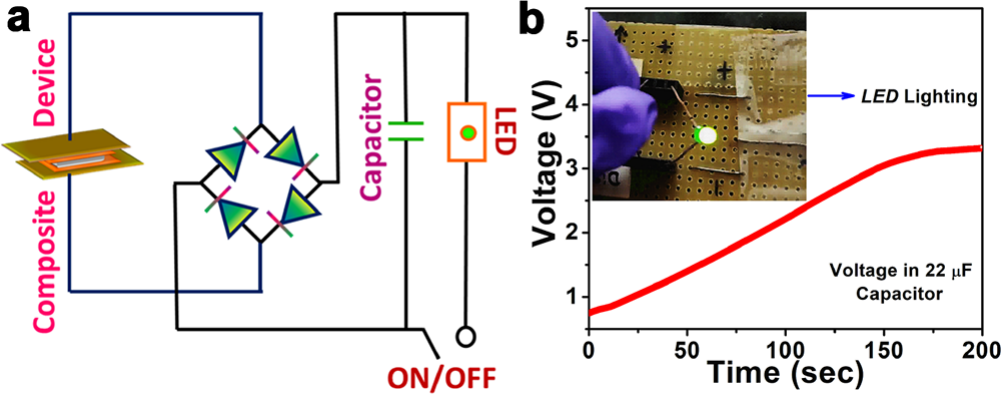
(a) The circuit diagram of the full-wave four-diode bridge rectifier circuit utilized for capacitor charging and LED lighting experiments. (b) The voltage accumulated in a 22 μF capacitor by utilizing the **3DP-*****Gy***. Inset: the image of the lighted green LED by using the charge stored in the 22 μF capacitor.

To assess the energy storage capabilities of the 10 wt % **(*****p*****-TEA)(*****p*****-TEAH)·PF**_**6**_-PCL and **3DP-*****Gy*** devices, capacitor charging experiments were conducted using capacitors with different capacitance values. The devices were connected to a four-diode bridge rectifier circuit to convert the AC output voltages generated during impact measurements into DC voltages (Figure 7a). Thus, from the charging curves of a 100 μF capacitor, the maximum charge and energy stored were found to be 226.0 and 133.5 μC and 257.0 and 89.6 μJ, respectively, by employing the **3DP-*****Gy*** and 10 wt % **(*****p*****-TEA)(*****p*****-TEAH)·PF**_**6**_-PCL devices (Figure S38). The corresponding voltages accumulated in the 100 μF capacitor during the charging process also show a similar trend with maximum voltages of 2.25 and 1.33 V, respectively, for the **3DP-*****Gy*** and 10 wt % **(*****p*****-TEA)(*****p*****-TEAH)·PF**_**6**_-PCL devices (Figure S39). The obtained results indicate that the large-scale 3D-printed device has efficient energy harvesting and storage capabilities, as evidenced by the increased charge accumulation in the capacitors while using the **3DP-*****Gy*** device. Notably, using a lower-rated 22 μF capacitor, a higher storage capacity of 3.4 V was achieved, which in turn was sufficient to flash-light a green LED (Figure 7b). The corresponding maximum charge and energy stored in the 22 μF were calculated to be 72.0 μC and 121.0 μJ, respectively (Figure S40).

## Conclusions

The development of organic ferroelectric materials for piezoelectric energy harvesting requires structural asymmetry, polar order, and long-term stability. In this work, the synthesized cocrystal **(*****p*****-TEA)(*****p*****-TEAH)·PF**_**6**_ exhibits these attributes by the respective presence of chiral ammonium cations, noncoordinating PF_6_^–^ anions and a neutral amine with additional H-bonding interactions. The *P–E* hysteresis loop measurements on **(*****p*****-TEA)(*****p*****-TEAH)·PF**_**6**_ gave a saturation polarization (*P*_s_) of 0.95 μC cm^–2^. Remarkably, **(*****p*****-TEA)(*****p*****-TEAH)·PF**_**6**_ shows a higher electrostrictive coefficient (*Q*_33_) value of 2.02 m^4^ C^–2^ compared to well-known PVDF and ceramic-based piezoelectric materials. The polymer composites of varying weight percentages were then prepared by combining **(*****p*****-TEA)(*****p*****-TEAH)·PF**_**6**_ with biodegradable nonpiezoelectric PCL polymer and studied for PENG applications. The optimal 10 wt % **(*****p*****-TEA)(*****p*****-TEAH)·PF**_**6**_-PCL device produced a maximum output voltage of 13 V and a PD of 104.2 μW cm^–3^. Furthermore, by utilizing the miniaturized 3D printing technique a gyroid-shaped device containing 10 wt % of **(*****p*****-TEA)(*****p*****-TEAH)·PF**_**6**_ (**3DP-*****Gy***) has been prepared, which not only retained the *V*_OC-PP_ of the thin-film-based device but also improved the overall output performance to 22.8 V (increase by 9.8 V) and lead to the improvement of the power density (118.5 μW cm^–3^). The applicability of both 10 wt % **(*****p*****-TEA)(*****p*****-TEAH)·PF**_**6**_**–PCL** and **3DP-*****Gy*** under extremely humid conditions (99% RH) was then validated, as no degradation in performance was noted even after exposure to 99% RH. The generated output voltages were used to charge different rating capacitors. The 3D-printed device was found to accumulate more charge than that of the thin film-based device. Also, the charge accumulated in a 22 μF capacitor was utilized for flash-lighting a green LED. These findings indicate that the integration of a stable all-organic material with a biodegradable polymer such as PCL and modern 3D printing techniques provides a strategy for the development of lightweight and heavy metal-free devices for applications in future wearable electronics, where the combination of eco-friendly materials and efficient energy harvesting capabilities are highly desired.

## Experimental Section

### Materials and Methods

Hexafuorophosphoric acid (HPF_6_), (*S*)-1-(*p*-tolyl)ethanamine, and PCL were purchased from Sigma-Aldrich and directly used in the reactions. The thermogravimetric analysis was performed in a dry nitrogen atmosphere by utilizing the PerkinElmer STA-6000 analyzer at a heating rate of 10 °C/min. Similarly, the DSC analysis was conducted in a TA Q20 differential scanning calorimeter with heating and cooling rates of 10 °C/min under a dry nitrogen atmosphere. For recording the nuclear magnetic resonance (NMR) spectra (^13^C{^1^H} NMR, 100.62 MHz; ^1^H NMR, 400.13 MHz) in CDCl_3_, a Bruker (400 MHz) spectrometer was utilized. The melting point (uncorrected) analyses were conducted by utilizing the Buchi M-560 setup. The FT-IR spectrum in the 400–4000 cm^–1^ range was performed by using the PerkinElmer spectrometer. For powder X-ray diffraction (PXRD) data collection in the 2θ range of 5 to 50°, the Bruker D8 ADVANCE X-ray diffractometer was used. The field-emission scanning electron microscopy (FE-SEM) analysis of all **(*****p*****-TEA)(*****p*****-TEAH)·PF**_**6**_-PCL composites was done by using the Zeiss Ultra Plus FE-SEM.

### XPS Analysis

The structural composition of **(*****p*****-TEA)(*****p*****-TEAH)·PF**_**6**_ was validated through XPS analysis by utilizing a Thermo Fisher Scientific instrument, UK Model K alpha+ spectrometer, using monochromatic Al Kα anode as an X-ray source (1486.6 eV) operating at a power of 72 W. Vacuum pressures of 1.2 × 10^–8^ mbar and 2 × 10^–9^ mbar were maintained in the sample loading and analyzer chambers, respectively. Data acquisition utilized microfocused X-ray sources with a spot size of 400 μm. The high-resolution survey scan was performed with a pass energy of 200 eV, while the distinct core-level spectra were captured using a 50 eV pass energy. During the spectral acquisition, charge compensation was maintained through Ar^+^ ion beams and ultralow energy coaxial electrons. The standard C 1s at binding energy 284.6 eV was used for final binding energy calibrations to determine the binding energies of various elements in the sample. The base pressure of the spectrometer was kept in excess of 5 × 10^–9^ mbar and 1 × 10^–7^ mbar throughout data collection with the active flood gun. With an instrument resolution of ±0.1 eV, raw data were processed using Avantage software, applying smart background subtraction for peak fitting.

### Single-Crystal X-ray Diffraction Analysis

The crystal structure of **(*****p*****-TEA)(*****p*****-TEAH)·PF**_**6**_ was solved from its single-crystal X-ray diffraction (SCXRD) data collected at 100 and 298 K on a Bruker Smart Apex Duo diffractometer with Mo Kα (λ = 0.71073 Å) radiation. The crystal structures at 100 and 298 K were then solved by using the direct method and were refined by utilizing full-matrix least-squares against *F*^2^, using the SHELXL-2014/7 program integrated into the Apex 3 software.^56^ The nonhydrogen atoms were refined anisotropically, and hydrogen atoms were modeled in geometric positions to their parent atoms.^57^ The structures were refined as a two-component racemic twin. In the asymmetric unit, two F atoms of the PF_6_ ion were positionally disordered over two sites and located in special positions with half-occupancies each. The TEAH and TEA moieties in **(*****p*****-TEA)(*****p*****-TEAH)·PF**_**6**_ cannot be distinguished crystallographically; hence, their protons are refined with partial occupancies to provide an accurate charge balance. Thus, H1A has a full occupancy (1.0), while H1B and H1C atoms are refined with 0.75 occupancies each. The DIAMOND-3.1 software was utilized to generate the structural illustrations of **(*****p*****-TEA)(*****p*****-TEAH)·PF**_**6**_.

### Hirshfeld Surface Analysis

The SCXRD crystallographic information file (CIF) of **(*****p*****-TEA)(*****p*****-TEAH)·PF**_**6**_ was utilized for Hirshfeld surface analysis on the Crystal Explorer 3.1 program, and the various types of interactions such as normalized contact distance (*d*_norm_), curvedness, and shape index present on the Hirshfeld surface were visualized. The resulting 3D color mapping images showed the diverse surface color mappings of compound **(*****p*****-TEA)(*****p*****-TEAH)·PF**_**6**_, with intense interactions represented by red, medium interactions by blue, and weak interactions by white color. The 2D fingerprint plot was generated by compiling the distances between the atoms closest to the Hirshfeld surface interior (*d*_i_) and exterior (*d*_e_). The various contours in the 2D fingerprint plot, represented by blue and gray colors, provide more insight into the different molecular interactions present in the **(*****p*****-TEA)(*****p*****-TEAH)·PF**_**6**_ molecule.

### Second Harmonic Generation Analysis

A Coherent Astrella Ti:Sapphire regenerative amplifier (RA) was used to generate femtosecond laser pulses at a repetition rate of 1 kHz for the Kurtz–Perry powder tests. These laser pulses were passed through a wavelength-tunable TOPAS-Prime Vis-NIR optical parametric amplifier (OPA) to obtain the desired wavelength of 800 nm. The laser beams were unfocused and had a 0.20 mJ cm^–2^ fluence at 800 nm. The SHG relative efficiency of **(*****p*****-TEA)(*****p*****-TEAH)·PF**_**6**_ was estimated using the Kurtz–Perry powder method, and the potassium dihydrogen phosphate (KDP) sample was used as the reference. Microcrystals of **(*****p*****-TEA)(*****p*****-TEAH)·PF**_**6**_ and KDP of size fraction of 250–177 μm were ground and sieved through an Aldrich mini-sieve set. At a 45° angle, the laser beam was directed at the samples and the diffused SHG spectra of **(*****p*****-TEA)(*****p*****-TEAH)·PF**_**6**_ and KDP were recorded by an Ocean Optics Flame T spectrograph after suppressing the scattered pumping radiation with a short-pass dielectric filter of 750 nm.

### Ferroelectric, Dielectric, and Piezoelectric Measurements

The ferroelectric properties of **(*****p*****-TEA)(*****p*****-TEAH)·PF**_**6**_ were analyzed by *P–E* hysteresis measurements conducted on its compacted disc samples of ∼8 mm diameter and ∼1.2 mm thickness electroded with Cu adhesive tapes as top and bottom contacts. The aixACCT TF 2000 E model hysteresis loop analyzer was utilized for the *P–E* hysteresis loop measurements. The dynamic leakage current compensation (DLCC) mode was applied to reduce the contributions from nonhysteretic components of the *P–E* loop. The ferroelectric fatigue measurements were performed for 10^6^ cycles under identical DLCC conditions. A frequency of 100 Hz and an applied voltage of 100 V were applied for conducting the ferroelectric fatigue measurements.

The Solartron Analytical Impedance Analyzer model 1260, coupled with a Dielectric Interface 1296A, was utilized for the dielectric permittivity and impedance measurements. The compacted pellet of **(*****p*****-TEA)(*****p*****-TEAH)·PF**_**6**_ was utilized for the dielectric measurements. The compacted pellet of **(*****p*****-TEA)(*****p*****-TEAH)·PF**_**6**_ was placed in a Janis 129610A cryostat sample holder, and the temperature accuracy was controlled by using a Lakeshore 336 model temperature controller.

The piezoelectric coefficient (*d*_33_) of a compacted disc (∼8 mm and thickness ∼1.2 mm) sample of **(*****p*****-TEA)(*****p*****-TEAH)·PF**_**6**_ was obtained from the Berlincourt (PM300) Piezotest meter.

### PFM Characterizations

The PFM analysis was conducted using the Asylum Research MFP-3D atomic force microscopy (AFM) system for a drop-casted thin film of **(*****p*****-TEA)(*****p*****-TEAH)·PF**_**6**_ on the indium tin oxide (ITO)-coated glass surface. A contact mode AFM experiment was carried out, utilizing RMN-12PT300B cantilever probes with a spring constant of 1.12 N m^–1^ and a tip diameter of less than 8 nm. Vertical-PFM experiments were employed to acquire PFM data, with an applied AC voltage to the conductive AFM tip, while the bottom electrode was grounded. At a resonance frequency of 300 ± 20 kHz with a varied applied bias of 60 and 80 V, the PFM images were collected. The measurements were performed using dual AC resonance tracking (DART) mode. The switching ability of the single-crystal domains located on the thin film was verified by the application of external DC bias of ±50 V using the contact mode PFM.

### General Procedure for the Preparation of Polymer Composite Films and Devices

To prepare the polymer composite films, varying amounts (5, 10, 15, and 20 wt %) of **(*****p*****-TEA)(*****p*****-TEAH)·PF**_**6**_ crystallites were dispersed in biodegradable nonpiezoelectric PCL polymer in chloroform (CHCl_3_) solution. The polymer composite solutions were mechanically stirred at 50 °C for 30 min and further vortex mixed for 15 min to obtain homogeneous solutions. These homogeneous composite solutions were then poured onto glass plates and allowed to dry at room temperature for 8 h. The free-standing **(*****p*****-TEA)(***p***-TEAH)·PF**_**6**_-PCL films were peeled off from the glass slide and copper with conductive adhesive tapes was attached to both sides for electrical contacts. The devices were then completely encapsulated with adhesive Kapton tapes for electrical insulation. Additionally, a PCL polymer film was also encapsulated with Kapton tape for comparison.

### General Procedure for the Preparation of Composite Filaments

The **(*****p*****-TEA)(*****p*****-TEAH)·PF**_**6**_-PCL composite filaments were produced through a two-step process involving solution mixing and melt extrusion. First, the prepared **(*****p*****-TEA)(*****p*****-TEAH)·PF**_**6**_-PCL composite films were shredded and melt compounded for 3 min in a HAAKE MiniCTW twin-screw extruder at 110 °C. The screw and take-up roller speeds were optimized to extrude filaments of 2.85 ± 0.15 mm diameter from the extruder die.

### Procedure for the Preparation of 3D-Printed Polymer Composite Devices

The prepared **(*****p*****-TEA)(*****p*****-TEAH)·PF**_**6**_-PCL composite filaments were dried in a vacuum oven at 25 °C for 24 h before 3D printing. An Ultimaker 3 FDM 3D printer was utilized to produce 3D structures with gyroid patterns. The printing parameters were optimized to achieve the best results for the **3DP-*Gy*** composites (Table S1).

### Piezoelectric Energy Harvesting and Storage Measurements

A custom-built periodic impact instrument operating at an impact force of 21 N and 2–10 Hz frequency was used to conduct the mechanical energy harvesting experiments. The Tektronix 2024 Mixed Signal Oscilloscope (input impedance ∼1 MΩ) was used to measure the open-circuit voltages and short-circuit currents. The devices under test had a thickness of approximately 0.5 mm and an active area of 360 mm^2^, while the 3D-printed **3DP-*****Gy*** devices had a thickness of about 0.9 mm and an area of 600 mm^2^. To test the energy storage attributes of the devices during impact measurements, different capacitors with varying capacitance values were used. For the capacitor charging experiments, the devices and capacitors were connected using a full-wave-bridge four-diode circuit. The flash-lighting of a green LED (power rating = 60 mW) was achieved by connecting the LED in series to the capacitor.

### Synthesis of **(*p*-TEA)(*p*-TEAH)·PF_6_**

To a flask containing (*S*)-*p*-tolylethanamine (0.3 g, 2.21 mmol), excess HPF_6_ (3.2 g, 22.1 mmol) was added slowly under constant stirring over a period of 10 min at room temperature. A white precipitate was formed, which was then dissolved again by adding methanol to obtain a clear solution. The filtered solution through a thick Celite pad was then kept at room temperature for crystallization. **(*****p*****-TEA)(*****p*****-TEAH)·PF**_**6**_ white crystals were obtained after a week. Yield: 85%. Anal. calcd for C_18_H_27_F_6_N_2_P: C 51.92; H 6.54; N 6.73. Found: C 51.02; H 6.23; N 6.70. Melting point: 490–500 K. ^1^H NMR (400 MHz, MeOD) δ 7.30 (dd, *J* = 41.7, 8.0 Hz, 4H), 4.43 (q, *J* = 6.8 Hz, 1H), 2.35 (s, 3H), 1.62 (d, *J* = 6.9 Hz, 3H). ^13^C NMR (101 MHz, MeOD) δ 138.67 (s), 135.50 (s), 129.35 (s), 126.26 (s), 50.67 (s), 19.73 (s), 19.30 (s). FTIR (cm^–1^): 3100, 1600, 1521, 1230, and 1016.

## Supporting Information Available

X-ray crystallographic data for **(*****p*****-TEA)(*****p*****-TEAH)·PF**_**6**_ at 100 and 298 K in the CIF format; additional figures pertaining to crystal structures, Hirshfeld Surface analysis, XPS analysis, PXRD, TGA, dielectric measurements, SEM, and tables of bond lengths, bond angles, and piezoelectric device fabrication and analyses (PDF)
Deposition numbers 2290334–2290335 (for **(*p*-TEA)(*p*-TEAH)·PF6** at 100 K) (CIF)
Deposition numbers 2290334–2290335 (for **(*p*-TEA)(*p*-TEAH)·PF6** at 298 K) (CIF)

## Supplementary Material

am4c03349_si_001.pdf

am4c03349_si_002.cif

am4c03349_si_003.cif
