# Supplementary material for: A Highly Electrostrictive Salt Cocrystal and the Piezoelectric Nanogenerator Application of Its 3D-Printed Polymer Composite
Source: ACS Appl Mater Interfaces. 2024 May 10;16(20):26406–16. doi: 10.1021/acsami.4c03349 (PMC11129113; doi:10.1021/acsami.4c03349)
Supplement: Supplementary file 1 — am4c03349_si_001.pdf [file am4c03349_si_001.pdf]

Supporting Information

A Highly Electrostrictive Salt Cocrystal and the Piezoelectric 2 Nanogenerator Application of Its 3D-Printed Polymer Composite

Supriya Sahoo,† Rishukumar Panday,† Premkumar Kothavade,§ Vijay Bhan Sharma,⊥ Anirudh Sowmiyanarayanan,# Balu Praveenkumar,# Jan K. Zaręba,*,∇ Dinesh Kabra,*,⊥ Kadhiravan Shanmuganathan,*,§,∥ Ramamoorthy Boomishankar*,†,‡

†Department of Chemistry Indian Institute of Science Education and Research, Pune, Dr. Homi Bhabha Road, Pune – 411008, India.

‡Centre for Energy Science, Indian Institute of Science Education and Research, Pune, Dr. Homi Bhabha Road, Pune – 411008, India

Email: boomi@iiserpune.ac.in

§Polymer Science and Engineering Division and Academy of Scientific and Innovative Research, CSIR-National Chemical Laboratory, Dr. Homi Bhabha Road, Pune – 411008, India

∥Academy of Scientific and Innovative Research (AcSIR), Ghaziabad 201002, India

Email: k.shanmuganathan@ncl.res.in

⊥Department of Physics and Center for Research in Nanotechnology and Sciences, Indian Institute of Technology, Mumbai 400076, India

Email: dkabra@iitb.ac.in

#PZT Centre, Armament Research and Development Establishment, Dr. Homi Bhabha Road, Pune – 411021, India

∇Institute of Advanced Materials, Wrocław University of Science and Technology, 50-370 Wrocław, Poland

Email: jan.zareba@pwr.edu.pl

Table of contents

S.No.	Details	Page No.
1	Synthesis, NMR, and X-ray crystallographic information	1-5
2	Hirshfeld surface analysis data and Characterizations	5-8
3	Dielectric Profiles	9
4	Preparation of **(*p*-TEA)(*p*-TEAH)·PF_6**-PCL composites, characterizations and their Piezoelectric Energy Harvesting studies	9-14
5	3D-printed **3DP-*Gy*** composites, their characterization and Piezoelectric Energy Harvesting studies	14-18
6	References	18

Table S1. The optimal printing conditions for **3DP-*Gy*** composite.

**Process Parameters**	**Values**
Bed	Glass plate
Nozzle size (mm)	0.4
Nozzle temperature (°C)	120
Bed temperature (°C)	30
Infill degree (%)	50
Layer height (mm)	0.1
Flow (%)	100
Print speed (mm/s)	30

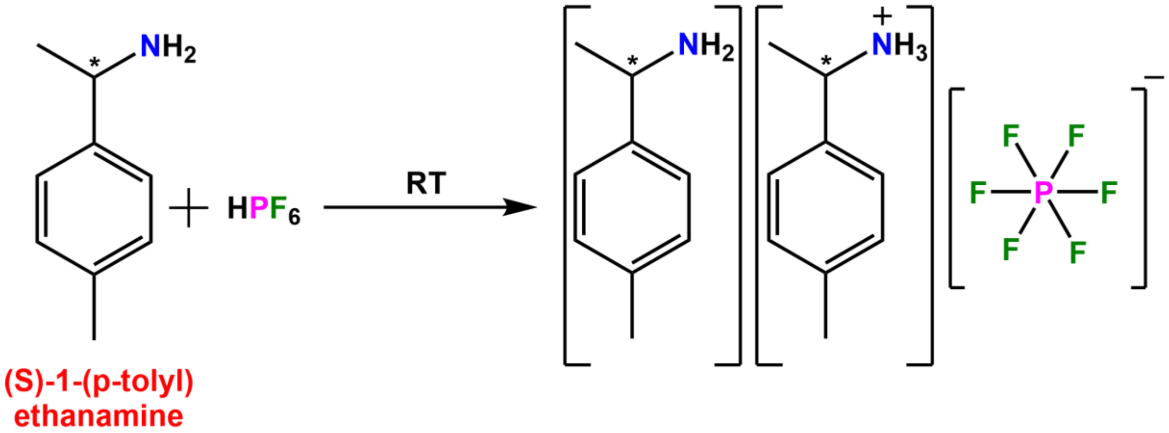

Scheme S1. Schematic showing the synthesis of **(*p*-TEA)(*p*-TEAH)·PF_6**.

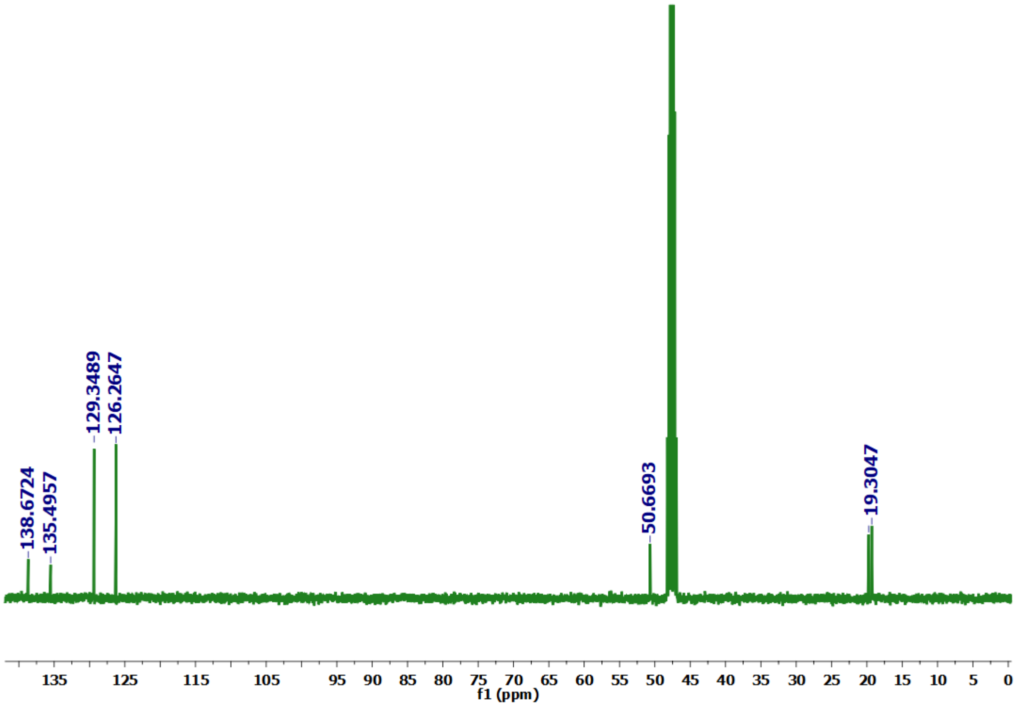

Figure S1. The ^{13}C-NMR Spectrum of **(*p*-TEA)(*p*-TEAH)·PF_6**.

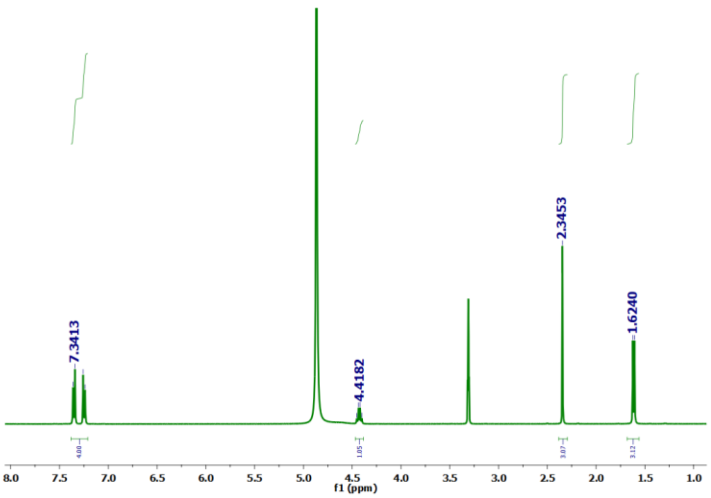

Figure S2. The ^{1}H-NMR Spectrum of **(*p*-TEA)(*p*-TEAH)·PF$_6$**.

Table S2. X-ray Crystallographic data for **(*p*-TEA)(*p*-TEAH)·PF$_6$**.

**Crystallographic details**	**100 K**	**298 K**
Chemical formula	$C_{18}H_{27}F_6N_2P$	$C_{18}H_{27}F_6N_2P$
Formula weight (g/mol)	416.39	416.39
Temperature	100(2)K	298(2)K
Crystal system	Monoclinic	Monoclinic
Space group	*C*2	*C*2
a (Å); α (°)	10.027(2); 90	10.131(2); 90
b (Å); β (°)	6.391(2); 104.41(3)	6.573(2); 103.48(3)
c (Å); γ (°)	16.343(3); 90	16.490(3); 90
V (Å^3); Z	1014.3(4); 2	1067.9(5); 2
ρ (calc.) g cm^{-3}	1.363	1.295
μ (Mo K$_\alpha$) mm^{-1}	0.194	0.184
2θ$_{max}$ (°)	50.002	50.948
R(int)	0.0844	0.0963
Completeness to θ	99.9	99.9
Data / param.	1775/145	1893/145
GOF	1.043	1.022
R1 [F>4σ(F)]	0.0440	0.0492
wR2 (all data)	0.1138	0.1223
max. peak/hole (e.Å^{-3})	0.320/-0.475	0.169/-0.262
Flack parameters	0.1(3)	0.2(3)

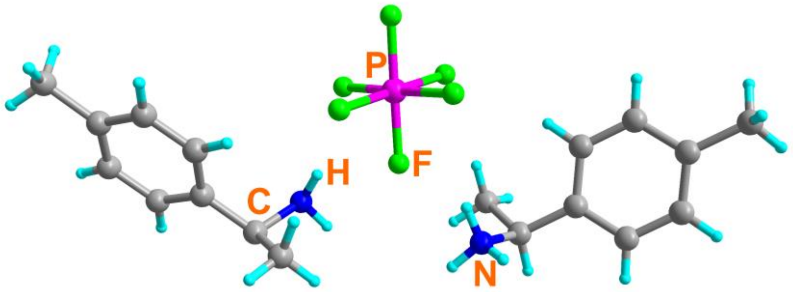

Figure S3. The molecular structure of **(*p*-TEA)(*p*-TEAH)·PF_6** at 298 K. The disordered F-atoms are omitted for clarity.

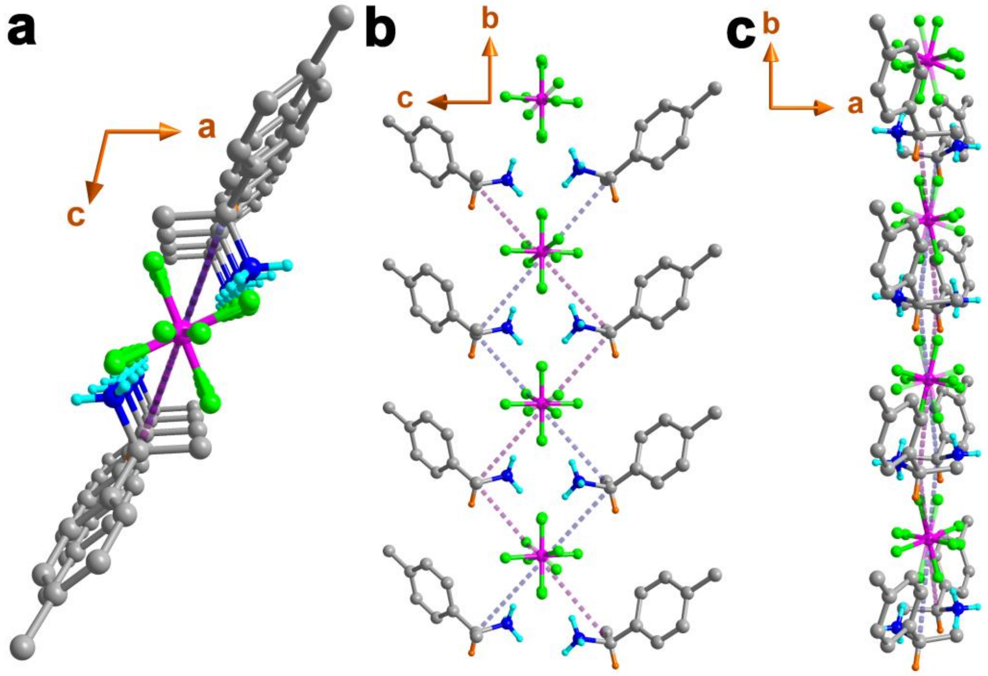

Figure S4. The packing diagram of **(*p*-TEA)(*p*-TEAH)·PF_6** along (a) *a*-axis, (b) *b*-axis (c) *c*-axis at 100 K including the disordered F-atoms.

Table S3. Hydrogen bonding parameters for **(*p*-TEA)(*p*-TEAH)·PF_6** at 100 K (without disorder).

D-H···A	d(H···A) Å	d(D-A) Å	<(DHA) °	Symmetry transformations to generate equivalent atoms
N(1)-H(1A)···F1	1.8394(21)Å	2.7401(38) Å	169.853(207) °	-1.5+x, -1.5+y, z
N(1)-H(1B)···F2	1.7576(83)Å	2.6116(95) Å	155.237(305) °	-1-x, -1+y, 1-z
N(1)-H(1C)···F3	1.7856(91)Å	2.5666(95) Å	142.274(298) °	-1+x, -2+y, z

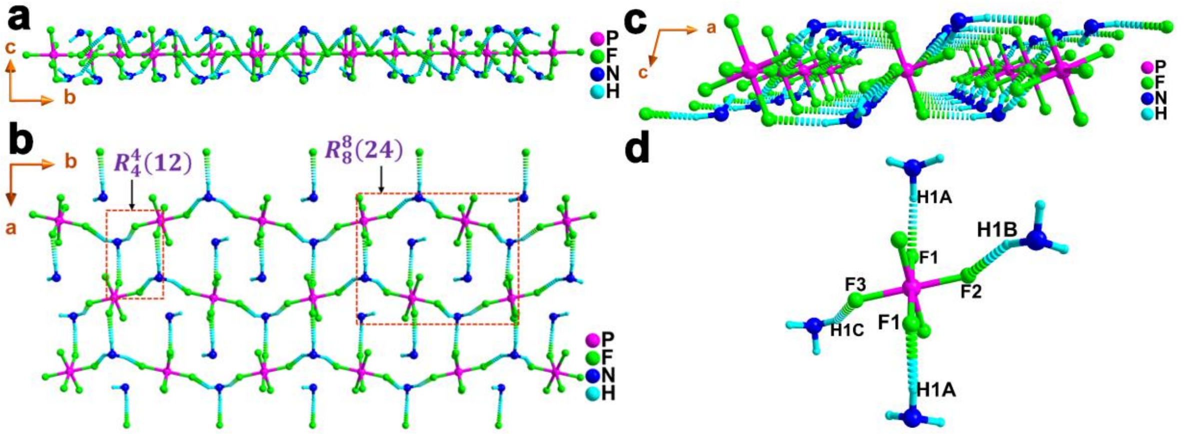

Figure S5. The N-H···F hydrogen bonding interactions in **(*p*-TEA)(*p*-TEAH)·PF_6** at 100 K. (a) along *a*-axis. (b) along *b*-axis. (c) along *c*-axis. (d) Repeating unit of the H-bonding sheet. C, H, and disordered F-atoms are omitted for clarity.

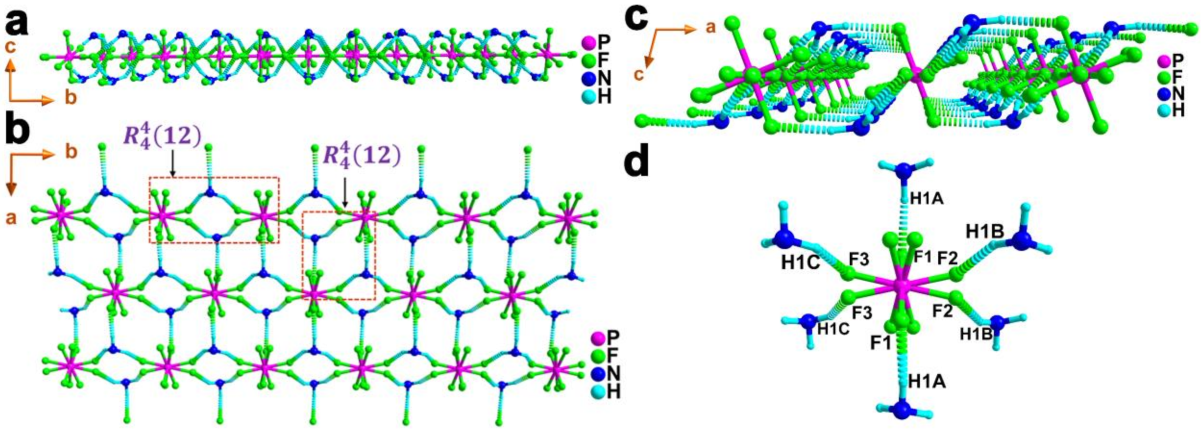

Figure S6. The N-H···F hydrogen bonding interactions in **(*p*-TEA)(*p*-TEAH)·PF_6** at 100 K, including the interactions from the disordered F-atoms. (a) along *a*-axis. (b) along *b*-axis. (c) along *c*-axis. (d) Repeating unit of the H-bonding sheet (C and H atoms are omitted for clarity).

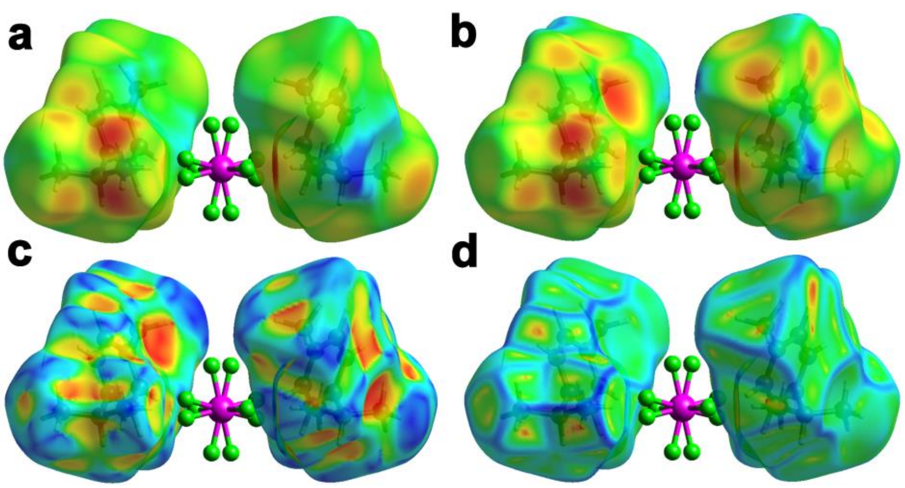

Figure S7. The 3D color mapping derived from the Hirshfeld surface analysis of **(*p*-TEA)(*p*-TEAH)·PF_6** (100 K) (including the interactions from the disordered F-atoms) showing (a) d_i, (b) d_e, (c) shape index and (d) curvedness.

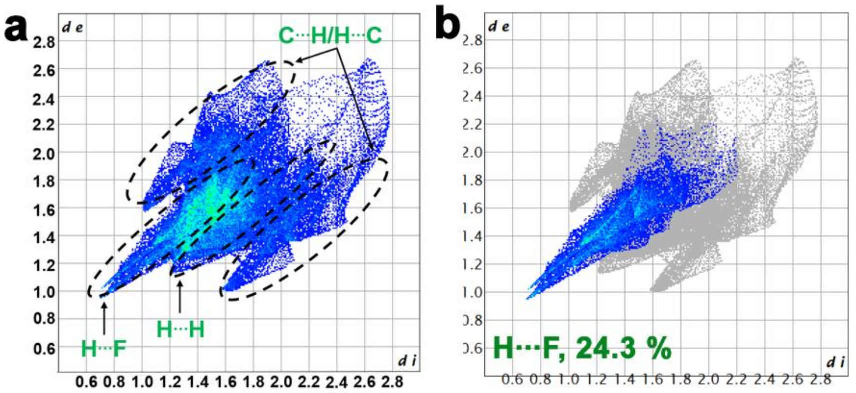

Figure S8. 2D fingerprint (d_e vs. d_i) plot of **(*p*-TEA)(*p*-TEAH)·PF$_6$** (100 K) showing the percentages of (a) all interactions and (b) H···F interactions in the molecule.

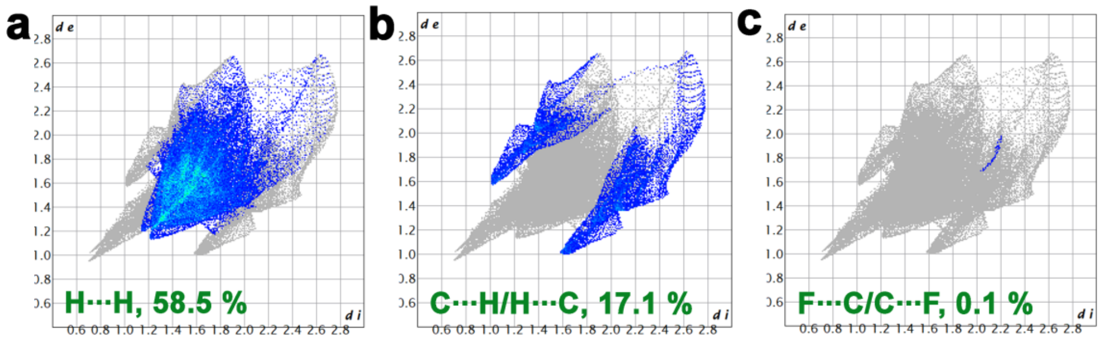

Figure S9. 2D fingerprint (d_e vs d_i) plot of **(*p*-TEA)(*p*-TEAH)·PF$_6$** (100 K) showing the percentages of (a) H···H, (b) C···H/H···C and (c) C···F/F···C interactions in the molecule.

Table S4. Percentage interactions present in **(*p*-TEA)(*p*-TEAH)·PF$_6$** at 100 K.

**Interactions**	**(*p*-TEA)(*p*-TEAH)·PF$_6$ (100 K) (%)**
H···F	24.3
H···H	58.5
C···H/H···C	17.1
C···F	0.1

Table S5. Hirshfeld surface analysis of **(*p*-TEA)(*p*-TEAH)·PF$_6$** at 100 K.

**Temperature**	**Surface Property**	**Range (Minimum/Maximum)**	**Globularity and Asphericity**	**Surface Volume and Area**
**(*p*-TEA)(*p*-TEAH)·PF$_6$ (100 K)**	d_i	0.7077/2.7844	0.653 and 0.349	411.03 Å^3 and 409.48 Å^2
	d_e	0.9618/2.7163		
	d_{norm}	-0.6960/1.5901		
	Shape index	-0.9952/0.9974		
	Curvedness	-3.3837/0.3661		

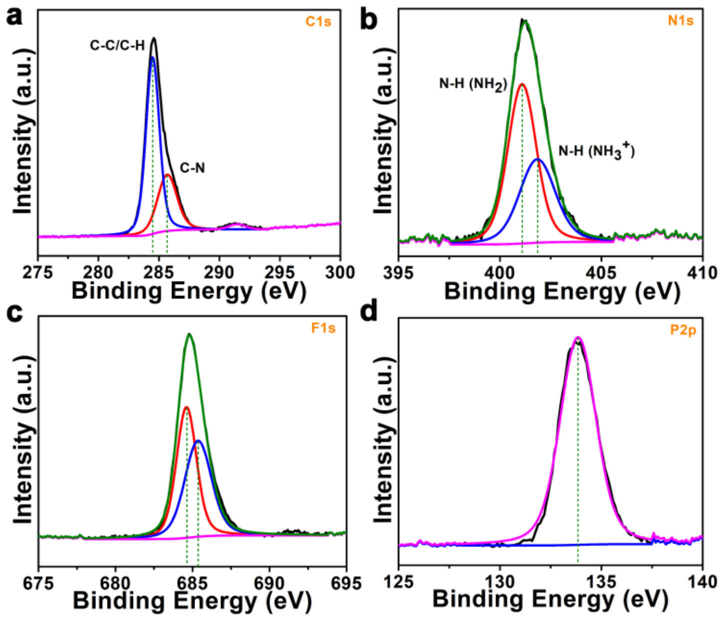

Figure S10. The X-ray photoelectron spectrums (a) C1s, (b) N1s, (c) F1s, and (d) P2p, of **(*p*-TEA)(*p*-TEAH)·PF_6**.

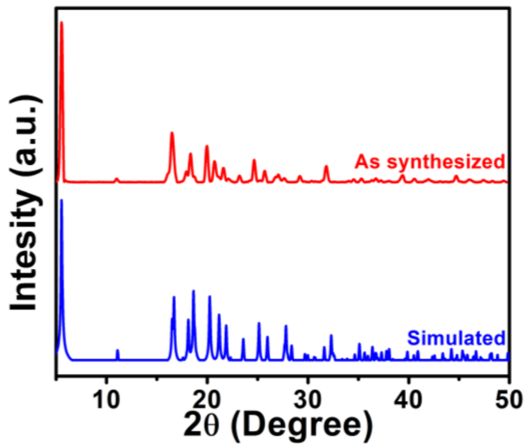

Figure S11. The room temperature PXRD profiles of **(*p*-TEA)(*p*-TEAH)·PF_6** along with its simulated profile from the 298 K single-crystal data.

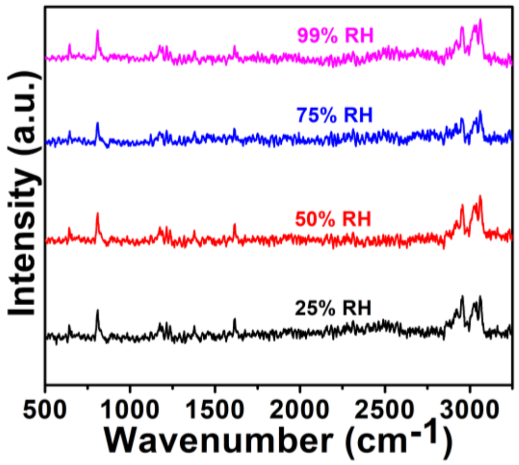

Figure S12. The Raman spectral profile of **(*p*-TEA)(*p*-TEAH)·PF_6** upon exposure to various humidity conditions showing its high stability.

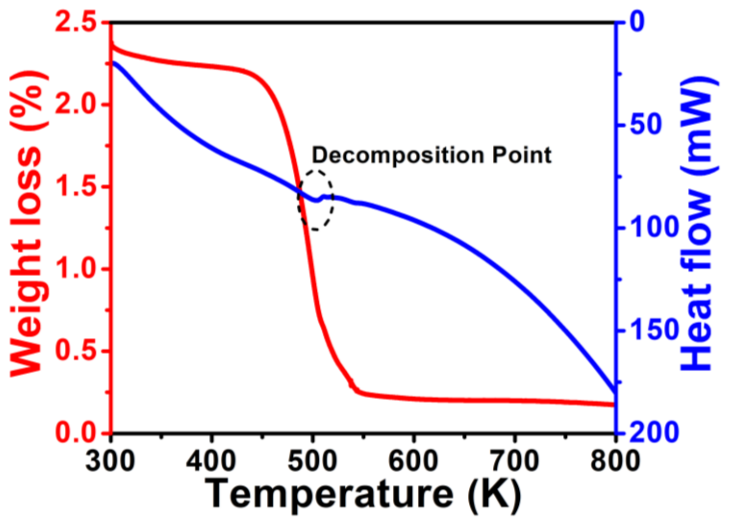

Figure S13. The thermogravimetric-differential thermal analysis profile of **(*p*-TEA)(*p*-TEAH)·PF_6.**

Table S6. Comparison of polarization and piezoelectric coefficient values of known organic ferroelectrics.

Ferroelectric Materials	Temperature (K)	Polarization	Piezoelectric Coefficient	References
Croconic acid	-	20 μC/cm^2	-	[1]
[3-oxoquinuclidinium]ClO_4	293	6.7μC/cm^2	-	[2]
4-(cyanomethyl)anilinium perchlorate	-	0.75 μC/cm^2	-	[2]
[Hdabco]ClO_4	-	4 μC/cm^2	-	[3]
thiourea	-	3.2 μC/cm^2	-	[2]
[H_2-TPPZ][Hba]$_2$	298	0.12 μC cm^{-2}	-	[4]
Im-CLO_4	293	7.5 μC cm^{-2}	41 pC N^{-1}	[5]
TFTBSA	298	0.87 μC cm^{-2}	-	[6]
MDABCO–NH_4I_3	373	19 μC/cm^2	14 pC N^{-1}	[7]
^{R}MBA-BF_3	298	4.27 μC cm^{-2}	3.5 pC N^{-1}	[8]
TPAP·BF_4	298	0.22 μC cm^{-2}	3 pC N^{-1}	[9]
TPAP·ClO_4	-	-	4 pC N^{-1}	[9]
DPDP·BF_4	298	5.36 μC cm^{-2}	7 pC N^{-1}	[9]
DPDP·ClO_4	298	21.83 μC cm^{-2}	12 pC N^{-1}	[9]
DPDP·IO_4	298	21.12 μC cm^{-2}	8 pC N^{-1}	[9]
TIAP·BF_4	-	-	3 pC N^{-1}	[9]
TIAP·ClO_4	-	-	3 pC N^{-1}	[9]
**(*p*-TEA)(*p*-TEAH)·PF_6**	**298**	**0.95 μC cm^{-2}**	**4 pC N^{-1}**	**This Work**

Note: TPPZ = 2,3,5,6-tetra(2′-pyridyl)pyrazine, Hba = bromanilate, TFTBSA = 3,4,5-trifluoro-*N*-(3,5-di-*tert*-butylsalicylidene)aniline, MDABCO = *N*-methyl-*N*′-diazabicyclo[2.2.2]octonium, ^{R}MBA = $^{R}C_6H_5CH(CH_3)NH_2BF_3$, TPAP = triphenylisopropylaminophosphonium, DPDP = diphenyl diisopropylaminophosphonium, TIAP = tetraisopropylaminophosphonium.

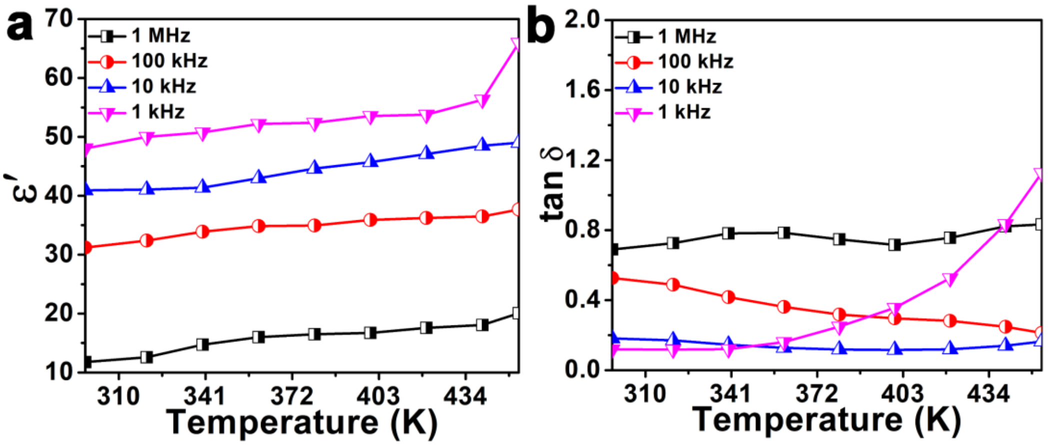

Figure S14. The temperature-dependant (a) dielectric permittivity and (b) dielectric loss plots of **(*p*-TEA)(*p*-TEAH)·PF$_6$**.

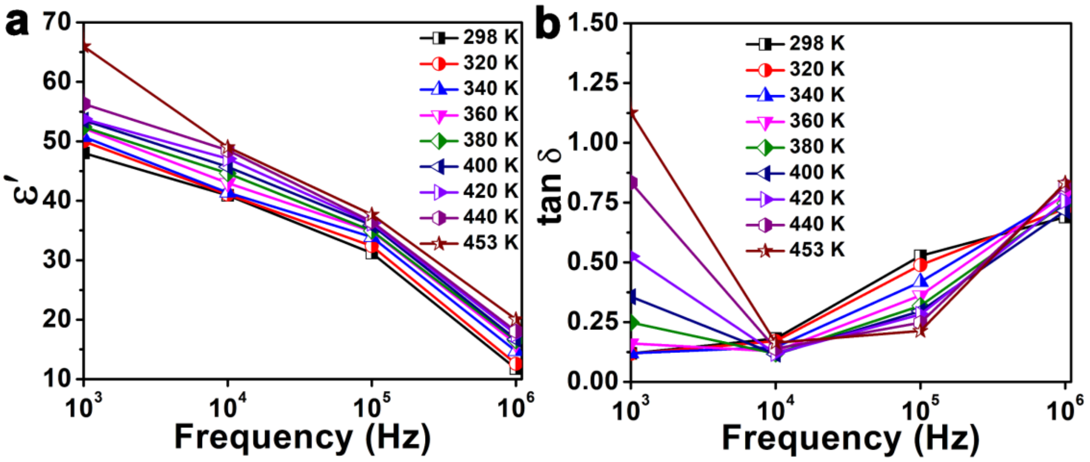

Figure S15. The frequency-dependant (a) dielectric permittivity and (b) dielectric loss plots of **(*p*-TEA)(*p*-TEAH)·PF$_6$**.

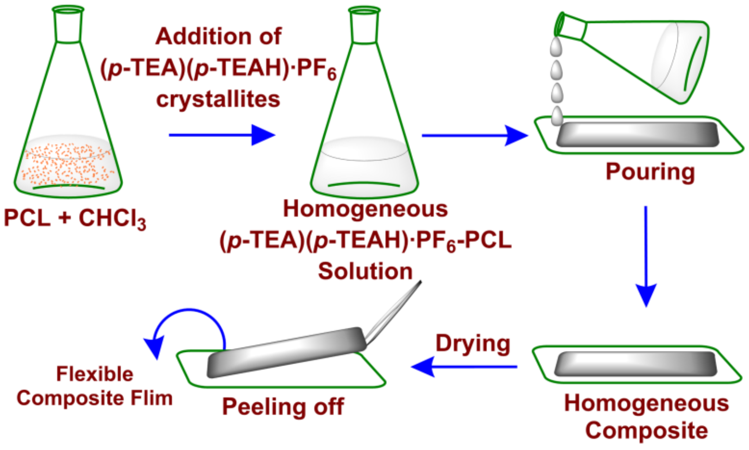

Figure S16. Schematic for the preparation of **(*p*-TEA)(*p*-TEAH)·PF$_6$**-PCL composite films.

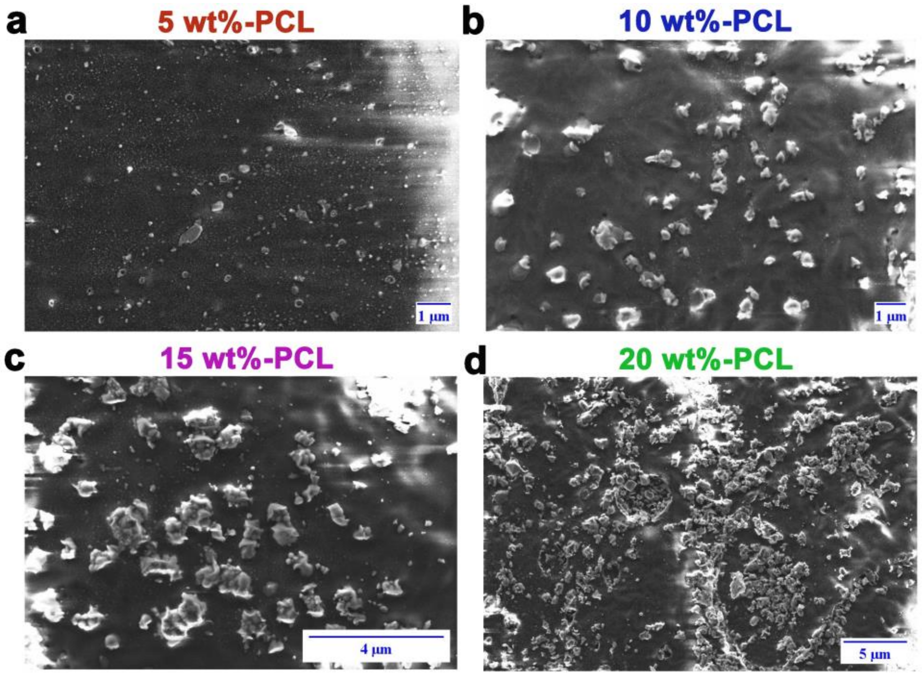

Figure S17. The FE-SEM images of (a) 5 wt%, (b) 10 wt%, (c) 15 wt% and (d) 20 wt% **(*p*-TEA)(*p*-TEAH)·PF₆**-PCL composites.

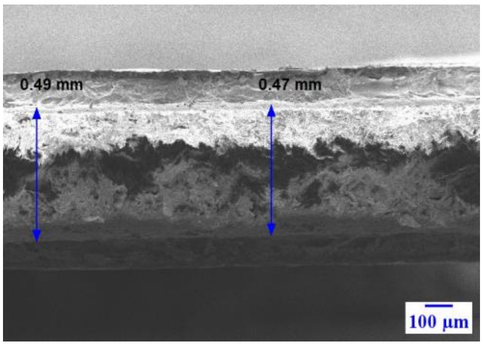

Figure S18. The FE-SEM cross-section image of the 10 wt% **(*p*-TEA)(*p*-TEAH)·PF₆**-PCL composite.

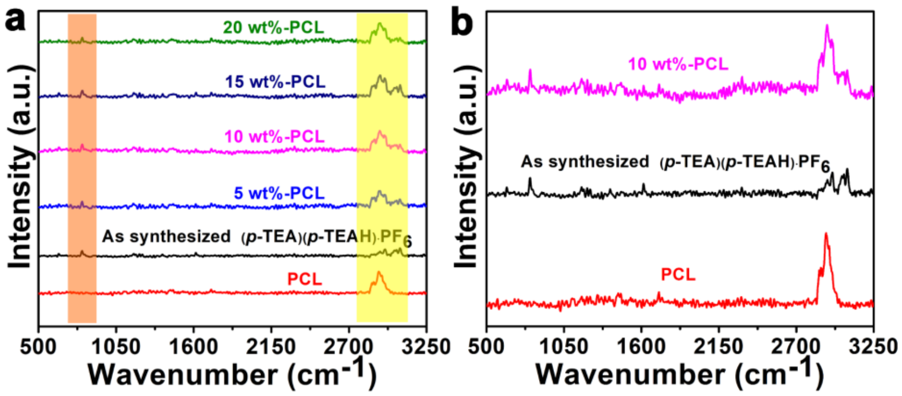

Figure S19. (a) The Raman spectral profile of all the wt% **(*p*-TEA)(*p*-TEAH)·PF₆**-PCL composite films compared to that of the neat compound **(*p*-TEA)(*p*-TEAH)·PF₆** and polymer PCL. (b) The zoomed-in Raman spectral profile of all the wt% **(*p*-TEA)(*p*-TEAH)·PF₆**-PCL composite films compared to that of the neat compound **(*p*-TEA)(*p*-TEAH)·PF₆** and polymer PCL.

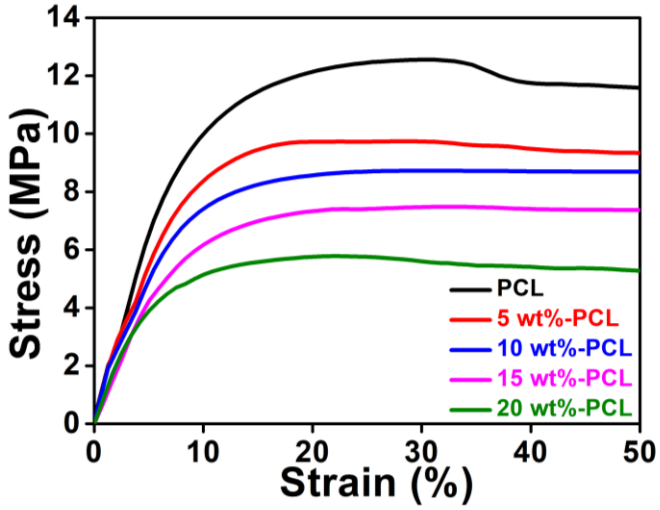

Figure S20. The stress-strain profile of PCL and **(*p*-TEA)(*p*-TEAH)·PF$_6$**-PCL composite films.

Table S7. The tensile toughness of neat PCL and **(*p*-TEA)(*p*-TEAH)·PF$_6$**-PCL composite films.

Composites	Tensile toughness (MJ/m^3)
PCL	146.8 ± 29
5 wt%-PCL	119.1 ± 22
10 wt%-PCL	90.4 ± 19
15 wt%-PCL	84.2 ± 17
20 wt%-PCL	70.8 ± 11

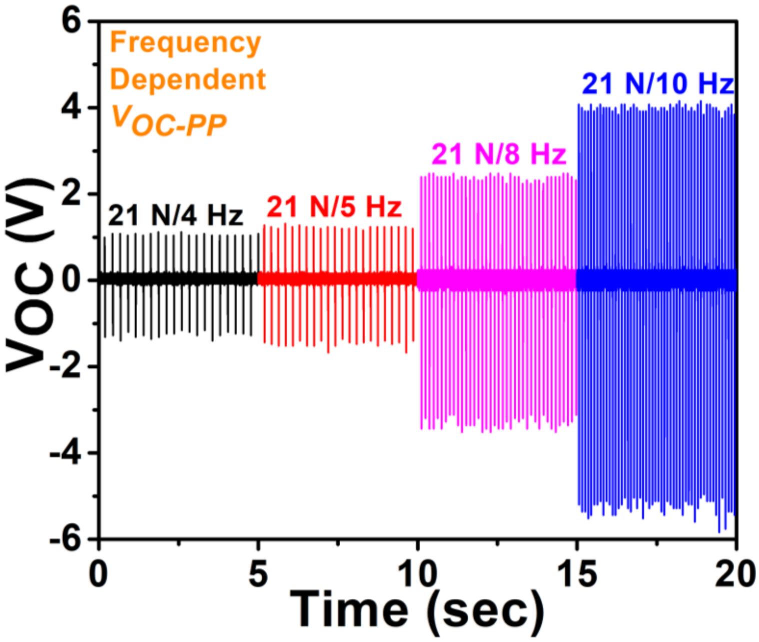

Figure S21. The frequency-dependent open-circuit peak-to-peak voltage ($V_{OC\text{-}PP}$) data of 10 wt% **(*p*-TEA)(*p*-TEAH)·PF$_6$**-PCL composite device.

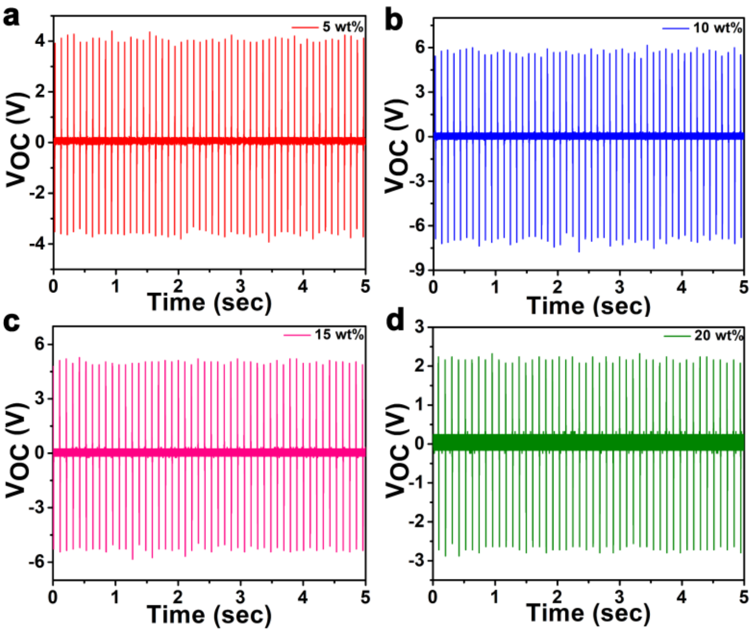

Figure S22. The open-circuit peak-to-peak voltage ($V_{OC\text{-}PP}$) profiles of (a) 5 wt%, (b) 10 wt%, (c) 15 wt% and (d) 20 wt% **(*p*-TEA)(*p*-TEAH)·PF$_6$**-PCL composite devices.

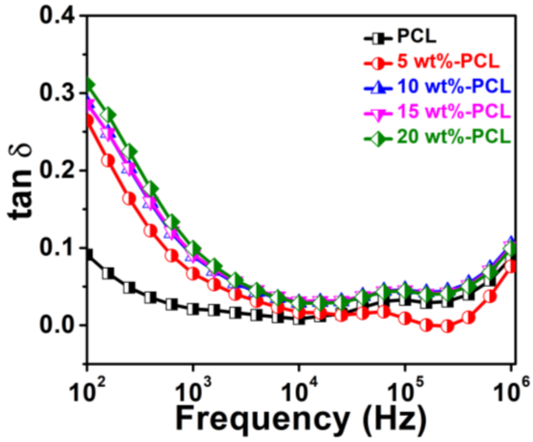

Figure S23. The frequency-dependent dielectric loss data for all **(*p*-TEA)(*p*-TEAH)·PF$_6$**-PCL composite films.

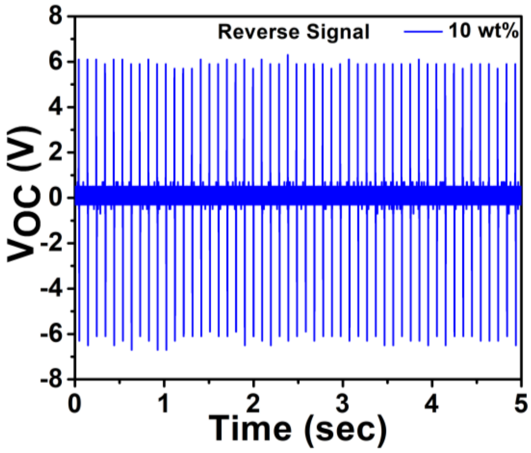

Figure S24. The open-circuit peak-to-peak voltage ($V_{OC\text{-}PP}$) obtained from of 10 wt% **(*p*-TEA)(*p*-TEAH)·PF$_6$**-PCL upon reversing the connections.

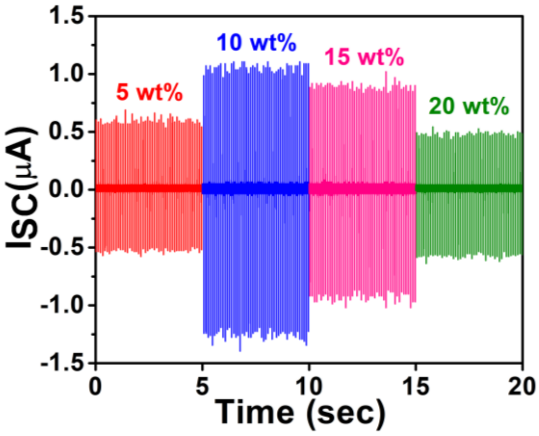

Figure S25. The output peak-to-peak current (I_{PP}) profile calculated from the voltage drop obtained by attaching a 4.7 MΩ resistor across the circuit for the **(*p*-TEA)(*p*-TEAH)·PF$_6$**-PCL composite devices.

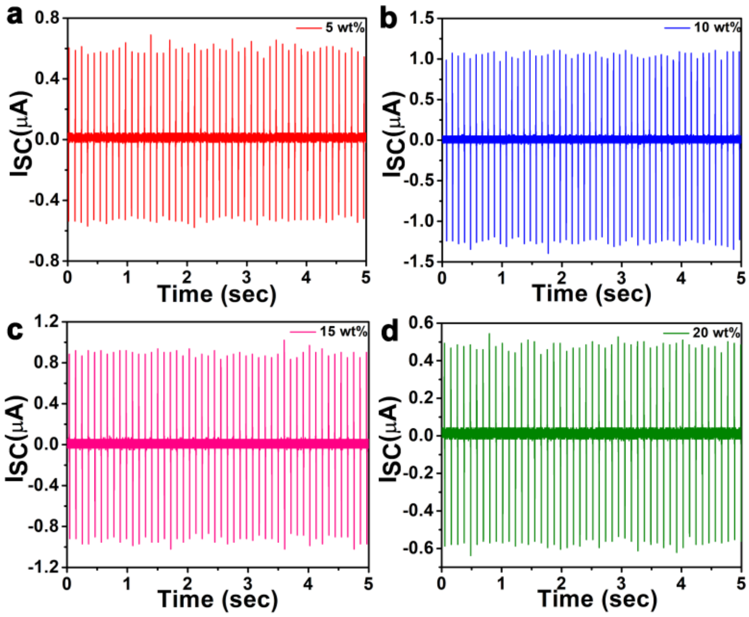

Figure S26. The output peak-to-peak current (I_{PP}) profile calculated from the voltage drop obtained by attaching a 4.7 MΩ resistor across the circuit for the (a) 5 wt%, (b) 10 wt%, (c) 15 wt% and (d) 20 wt% **(*p*-TEA)(*p*-TEAH)·PF$_6$**-PCL composite devices.

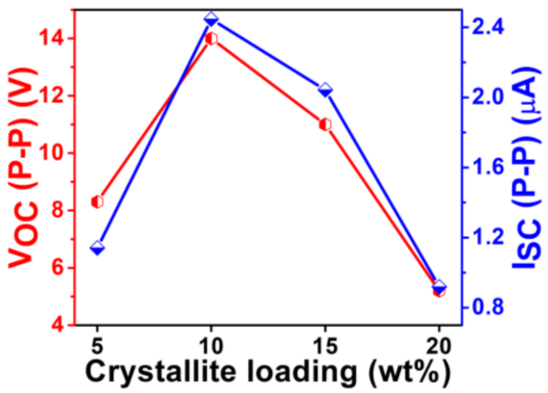

Figure S27. Comparative diagram showing the observed trends in $V_{OC\text{-}PP}$ and I_{PP} values of **(*p*-TEA)(*p*-TEAH)·PF$_6$**-PCL composite devices.

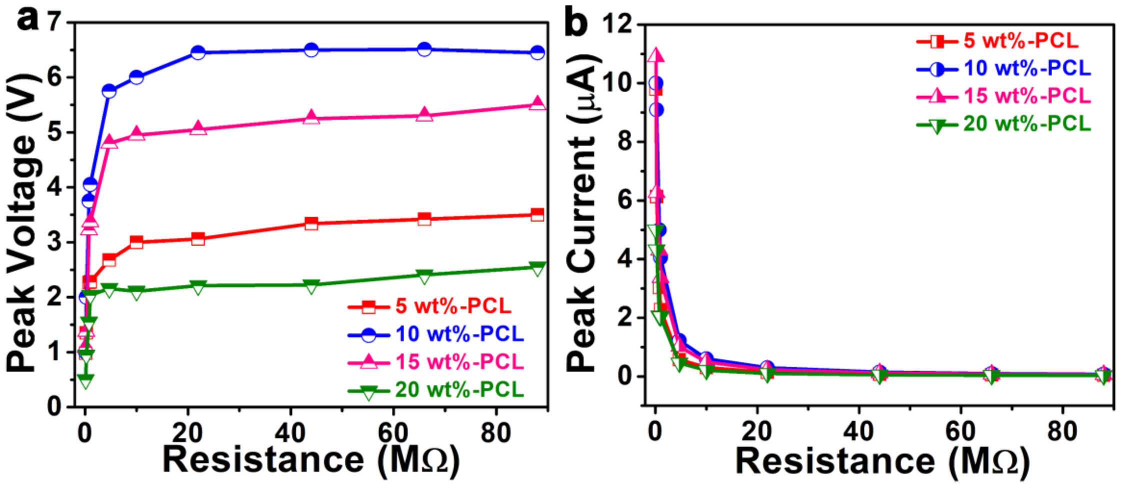

Figure S28. The comparative peak (a) voltage drop and (b) current data for all the **(*p*-TEA)(*p*-TEAH)·PF_6**-PCL composite devices under various load resistances (by default, the voltage and current generated during the compression cycles).

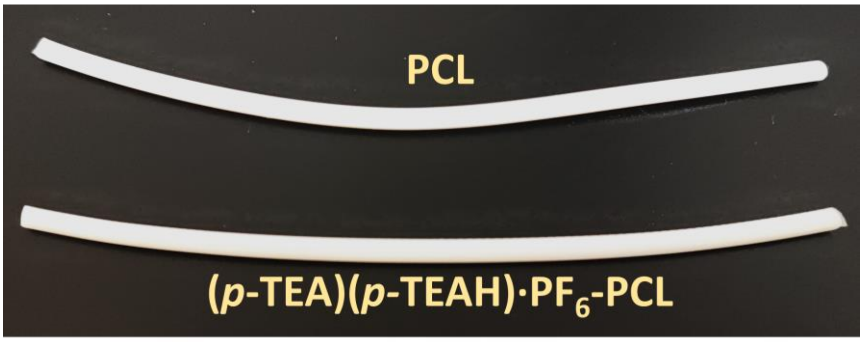

Figure S29. Pictures of the raw filaments.

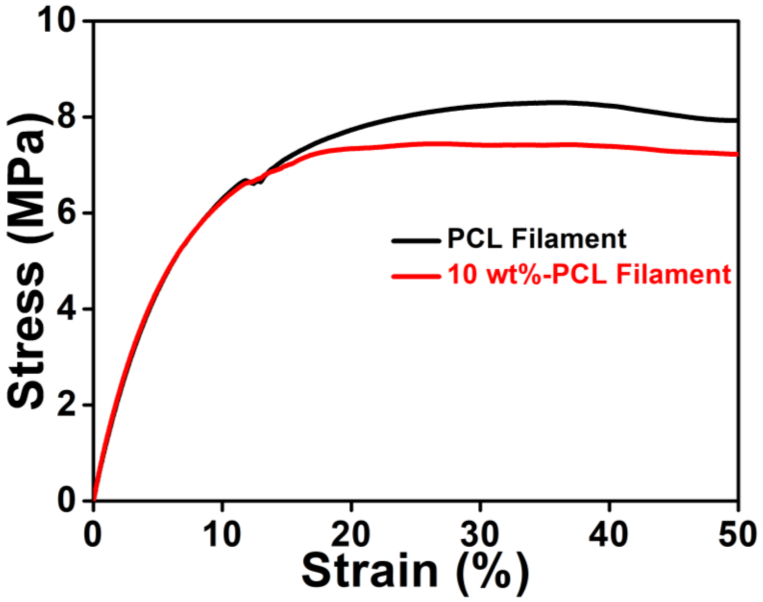

Figure S30. The stress-strain profile of PCL and **(*p*-TEA)(*p*-TEAH)·PF_6**-PCL composite filaments.

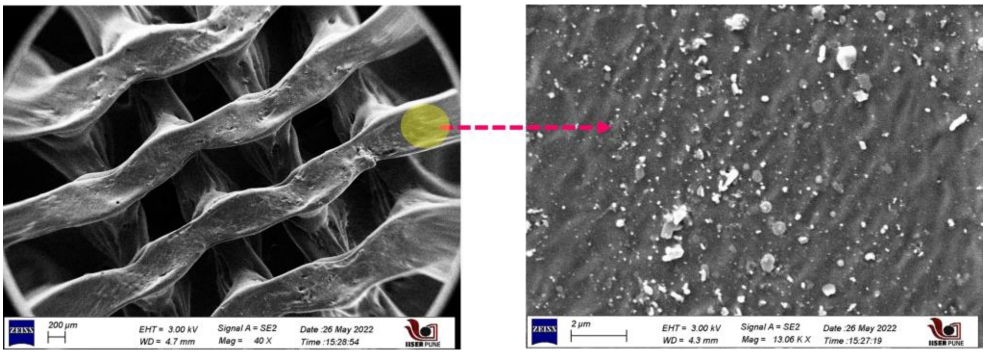

Figure S31. SEM images of 10 wt% **3DP-*Gy*** composite.

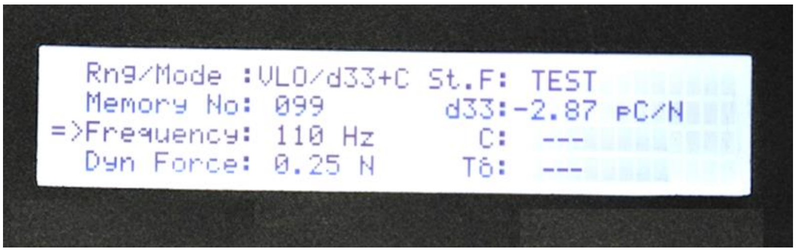

Figure S32. d_{33} value for the **3DP-*Gy*** slab.

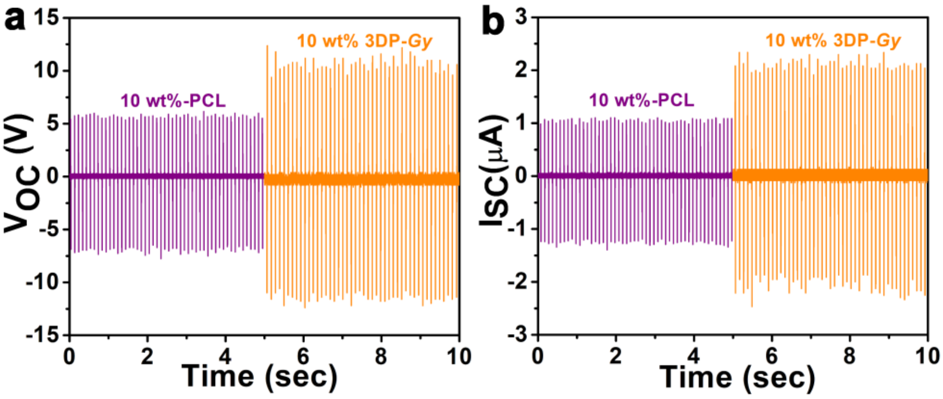

Figure S33. The comparative peak-to-peak (a) open-circuit voltage ($V_{OC\text{-}PP}$) and (b) calculated current (I_{PP}) profiles of 3D printed 10 wt% **3DP-*Gy*** to that of 10 wt% **(*p*-TEA)(*p*-TEAH)·PF$_6$**-PCL thin film.

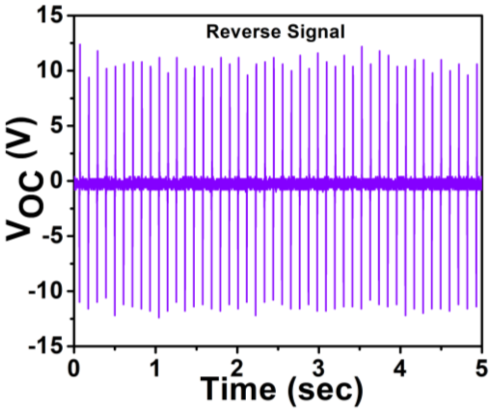

Figure S34. The open-circuit peak-to-peak voltage ($V_{OC\text{-}PP}$) obtained from of 10 wt% **3DP-*Gy*** upon reversing the connections.

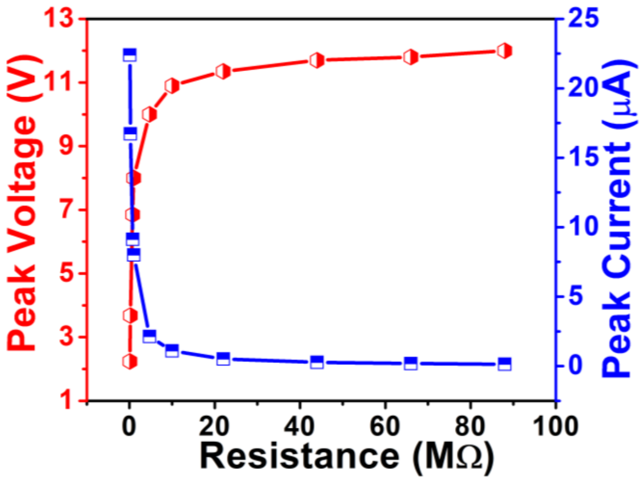

Figure S35. The comparative peak voltage drops and current data for 10 wt% **3DP-*Gy*** composite device under various load resistances (by default, the voltage and current generated during the compression cycles).

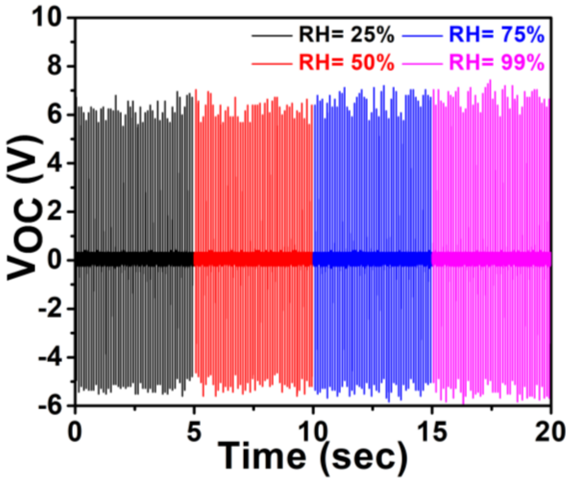

Figure S36. The RH dependent open-circuit peak-to-peak voltage ($V_{OC\text{-}PP}$) of 10 wt% **(*p*-TEA)(*p*-TEAH)·PF$_6$**-PCL device.

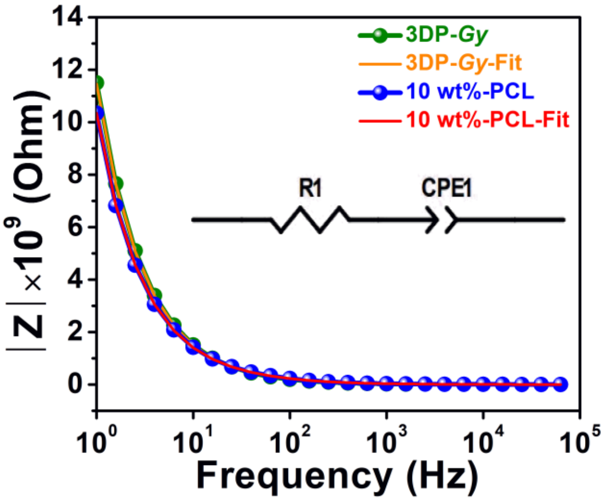

Figure 37. Frequency-dependent total impedance (*Z*) for **3DP-*Gy*** slab and 10 wt% **(*p*-TEA)(*p*-TEAH)·PF$_6$**-PCL composite.

Table S8. Impedance data fitting parameters for **3DP-*Gy*** slab and 10 wt% **(*p*-TEA)(*p*-TEAH)·PF_6**-PCL composite.

**Element**	**3DP-*Gy***	10 wt% **(*p*-TEA)(*p*-TEAH)·PF_6**-PCL
R1	-1.5805E05	-1.6139E05
CPE1-T	1.7187E-11	1.7149E-11
CPE1-P	0.88443	0.88339
Chi-Squared Value	0.012889	0.012962

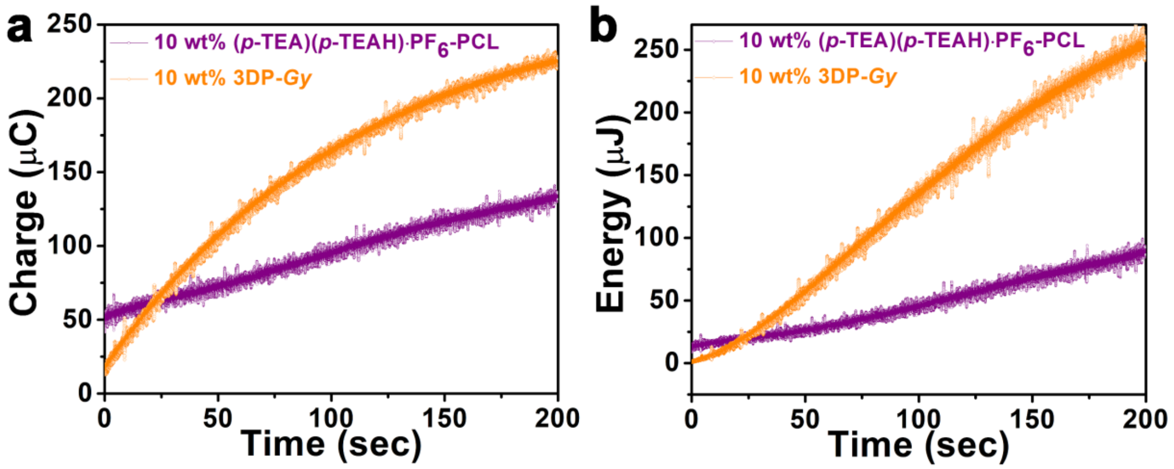

Figure S38. The (a) charges stored and (b) energies stored in a 100 µF capacitor by using the 10 wt% **3DP-*Gy*** and 10 wt% **(*p*-TEA)(*p*-TEAH)·PF_6**-PCL devices.

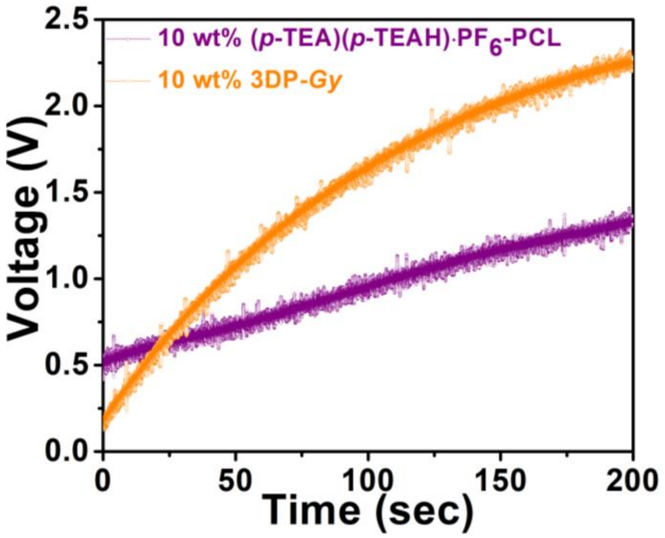

Figure S39. The voltages stored in a 100 µF capacitor by using the 10 wt% **3DP-*Gy*** and 10 wt% **(*p*-TEA)(*p*-TEAH)·PF_6**-PCL devices.

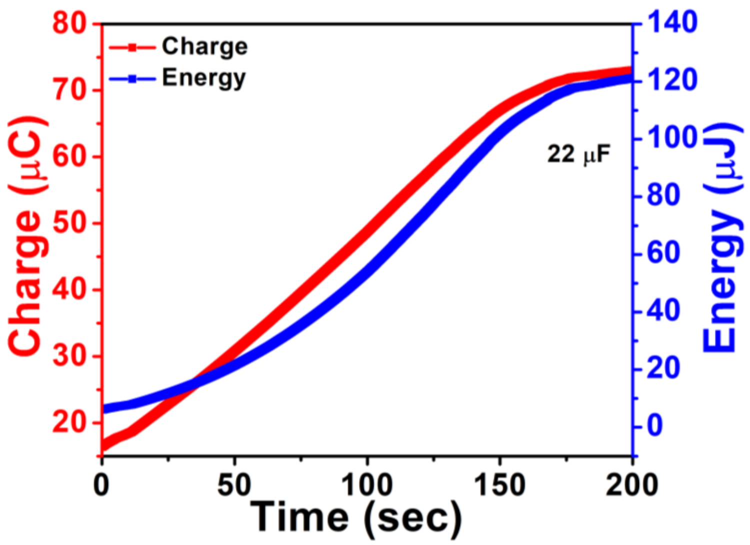

Figure S40. The charge and energy stored in a 22 μF capacitor by using the 10 wt% ***3DP-Gy*** device.

References:

1. Horiuchi, S.; Tokunaga, Y.; Giovannetti, G.; Picozzi, S.; Itoh, H.; Shimano, R.; Kumai, R.; Tokura, Y., Above-room-temperature ferroelectricity in a single-component molecular crystal. *Nature* **2010**, *463*, 789-792.
2. Yang, C.-K.; Chen, W.-N.; Ding, Y.-T.; Wang, J.; Rao, Y.; Liao, W.-Q.; Xie, Y.; Zou, W.; Xiong, R.-G., Directional intermolecular interactions for precise molecular design of a high-T_c multiaxial molecular ferroelectric. *J. Am. Chem. Soc.* **2019**, *141*, 1781-1787.
3. Tang, Y.-Y.; Zhang, W.-Y.; Li, P.-F.; Ye, H.-Y.; You, Y.-M.; Xiong, R.-G., Ultrafast polarization switching in a biaxial molecular ferroelectric thin film:[Hdabco]ClO_4. *J. Am. Chem. Soc.* **2016**, *138*, 15784-15789.
4. Horiuchi, S.; Kumai, R.; Tokunaga, Y.; Tokura, Y., Proton dynamics and room-temperature ferroelectricity in anilate salts with a proton sponge. *J. Am. Chem. Soc.* **2008**, *130*, 13382-13391.
5. Zhang, Y.; Liu, Y.; Ye, H.-Y.; Fu, D.-W.; Gao, W.; Ma, H.; Liu, Z.; Liu, Y.; Zhang, W.; Li, J.; Yuan, G.-L.; Xiong, R.-G., A Molecular Ferroelectric Thin Film of Imidazolium Perchlorate That Shows Superior Electromechanical Coupling. *Angew. Chem. Int. Ed.* **2014**, *53*, 5064-5068.
6. Tang, Y.-Y.; Liu, J.-C.; Zeng, Y.-L.; Peng, H.; Huang, X.-Q.; Yang, M.-J.; Xiong, R.-G., Optical control of polarization switching in a single-component organic ferroelectric crystal. *J. Am. Chem. Soc.* **2021**, *143*, 13816-13823.
7. Ye, H.-Y.; Tang, Y.-Y.; Li, P.-F.; Liao, W.-Q.; Gao, J.-X.; Hua, X.-N.; Cai, H.; Shi, P.-P.; You, Y.-M.; Xiong, R.-G., Metal-free three-dimensional perovskite ferroelectrics. *Science* **2018**, *361*, 151-155.
8. Sahoo, S.; Mukherjee, S.; Sharma, V. B.; Hernández, W. I.; Garcia-Castro, A. C.; Zaręba, J. K.; Kabra, D.; Vaitheeswaran, G.; Boomishankar, R., A Chiral B−N Adduct as a New Frontier in Ferroelectrics and Piezoelectric Energy Harvesting. *Angew. Chem. Int. Ed.* **2024**, e202400366.
9. Vijayakanth, T.; Ram, F.; Praveenkumar, B.; Shanmuganathan, K.; Boomishankar, R., All-Organic Composites of Ferro- and Piezoelectric Phosphonium Salts for Mechanical Energy Harvesting Application. *Chem. Mater.* **2019**, *31*, 5964-5972.
